# A New Taxon of Basal Ceratopsian from China and the Early Evolution of Ceratopsia

**DOI:** 10.1371/journal.pone.0143369

**Published:** 2015-12-09

**Authors:** Fenglu Han, Catherine A. Forster, James M. Clark, Xing Xu

**Affiliations:** 1 School of Earth Sciences, China University of Geosciences, Wuhan, China; 2 Key Laboratory of Evolutionary Systematics of Vertebrates, Institute of Vertebrate Paleontology and Paleoanthropology, Chinese Academy of Sciences, Beijing, China; 3 Department of Biological Sciences, The George Washington University, Washington, District of Columbia, United States of America; University of Akron, UNITED STATES

## Abstract

Ceratopsia is one of the best studied herbivorous ornithischian clades, but the early evolution of Ceratopsia, including the placement of *Psittacosaurus*, is still controversial and unclear. Here, we report a second basal ceratopsian, *Hualianceratops wucaiwanensis* gen. et sp. nov., from the Upper Jurassic (Oxfordian) Shishugou Formation of the Junggar Basin, northwestern China. This new taxon is characterized by a prominent caudodorsal process on the subtemporal ramus of the jugal, a robust quadrate with an expansive quadratojugal facet, a prominent notch near the ventral region of the quadrate, a deep and short dentary, and strongly rugose texturing on the lateral surface of the dentary. *Hualianceratops* shares several derived characters with both *Psittacosaurus* and the basal ceratopsians *Yinlong*, *Chaoyangsaurus*, and *Xuanhuaceratops*. A new comprehensive phylogeny of ceratopsians weakly supports both *Yinlong* and *Hualianceratops* as chaoyangsaurids (along with *Chaoyangsaurus* and *Xuanhuaceratops*), as well as the monophyly of Chaoyangosauridae + *Psittacosaurus*. This analysis also weakly supports the novel hypothesis that Chaoyangsauridae + *Psittacosaurus* is the sister group to the rest of Neoceratopsia, suggesting a basal split between these clades before the Late Jurassic. This phylogeny and the earliest Late Jurassic age of *Yinlong* and *Hualianceratops* imply that at least five ceratopsian lineages (*Yinlong*, *Hualianceratops*, *Chaoyangsaurus* + *Xuanhuaceratops*, *Psittacosaurus*, Neoceratopsia) were present at the beginning of the Late Jurassic.

## Introduction

Many Asian ceratopsians have helped clarify the early evolution of Ceratopsia in recent years, including *Yinlong downsi* [1], *Chaoyangsaurus youngi* [2], *Xuanhuaceratops niei* [3], *Liaoceratops yanzigouensis* [4], *Archaeoceratops oshimai* [5], *Archaeoceratops yujingziensis* [6], *Auroraceratops rugosus [7]*, and *Yamaceratops dorngobiensis* [8]. Recently, a basal neoceratopsian, *Aquilops americanus*, was discovered in the Lower Cretaceous Cloverly Formation of North America, suggesting an early migration from Asia into North America [9]. Recent phylogenetic analyses still debate the origin and early evolution of Ceratopsia. Some researchers suggest that *Yinlong* represents the most basal ceratopsian [1, 10], whereas others support a basal split between *Psittacosaurus* and the rest of Ceratopsia during the Jurassic [11]. Therefore, all new basal ceratopsian materials from Jurassic-age strata are potentially critical for clarifying the origin and early evolution of ceratopsians.

The earliest known ceratopsians are all from the Upper Jurassic of China, including *Chaoyangsaurus* from the Tuchengzi Formation of Liaoning province [2], *Xuanhuaceratops* from the Houcheng Formation of Liaoning province [3] and *Yinlong* from the Shishugou Formation, Wucaiwan locality, of Xinjiang province [1]. However, *Chaoyangsaurus* and *Xuanhuaceratops* are poorly preserved and their ages are still controversial [12, 13], making *Yinlong* the only unquestioned Jurassic ceratopsian. *Yinlong* also represents the most completely known of these Late Jurassic ceratopsians.

From 2001–2012 joint fieldwork by the Institute of Vertebrate Paleontology and Paleoanthropology and George Washington University has discovered many vertebrate remains, including more than 30 partial to nearly complete individuals of *Yinlong* [14]. Here we describe a new contemporaneous genus of basal ceratopsian, *Hualianceratops*, based mainly on skull material, and provide a phylogenetic analysis of Ceratopsia.

### Institutional Abbreviations

**IGCAGS**, Institute of Geology Chinese Academy of Geosciences, Beijing, China; **IVPP**, Institute of Vertebrate Paleontology and Paleoanthropology, Beijing, China.

## Materials and Methods

The specimen described here was jointly collected by the Institute of Vertebrate Paleontology and Paleoanthropology (IVPP) and George Washington University in 2002, and is catalogued in the collections of IVPP. According to the legislation of the People’s Republic of China, all necessary permits were obtained for the described field studies from Ministry of Land and Resources of the People's Republic of China and Department of Land and Resources of Xinjiang Uygur Autonomous Region, which complied with all relevant regulations as well as the PLoS Paleontological Ethics Statement.

### Phylogenetic analysis

To assess the systematic position of *Hualianceratops*, a new character list and matrix was compiled and analyzed (S1 and S2 Files). Our data matrix was mainly based on those of Ryan et al. [15] and Farke et al. [9], which were in turn modified from the matrices of Makovicky and Norell [8], Makovicky [16], Xu et al. [17], and Lee et al. [18]. However, these matrices contain few characters germane to the basalmost ceratopsians. To rectify this situation, characters were added from the matrices of Xu et al. [1] and Butler et al. [19]. Additionally, 19 new characters were added based on personal observation of basal ceratopsians, and four new taxa (*Aquilops*, *Auroraceratops*, *Psittacosaurus lujiatunensis*, *Yinlong wucaiwanensi*s) were coded into the matrix. The character coding for *Auroraceratops* and *Aquilops* were based on Morschhauser [11] and Farke et al. [9], respectively. *Psittacosaurus lujiatunensis* was coded from Zhou et al. [20], as well as the complete adult skull IVPP V12617 [21]. The codings for *Yinlong*, *Archaeoceratops*, *Liaoceratops*, *Xuanhuaceratops*, and *Chaoyangsaurus* were also modified where needed from previous analyses based on first hand observation (see S1 for details).

The final data matrix consists of 210 characters scored for 27 ingroup taxa. Ten outgroups were chosen to accurately polarize characters and determine the composition of Ceratopsia. The outgroup taxa include the basal ornithischians *Lesothosaurus*, *Heterodontosaurus*, and *Agilisaurus*, the non-iguanodontian ornithopods *Haya*, *Orodromeus* and *Jeholosaurus*, and the pachycephalosaurians *Wannanosaurus*, *Goyocephale*, *Homalocephale* and *Stegoceras*. The matrix was analyzed using TNT [22], and all characters were treated as equally weighted. Nine characters (19, 20, 70, 98, 128, 146, 171, 174, 178; characters 18, 19, 69, 97, 127, 145, 170, 173 and 177 in TNT’s protocol starting with state 0) were treated as ordered (additive). The analysis was conducted with the maximum trees set to 99,999 and zero-length branches collapsed, using a heuristic search with 1000 replicates of Tree Bisection and Reconnection (TBR) holding 10 trees with each replicate, followed by tree swapping using TBR on the trees in memory. Standard bootstrap values (absolute frequencies) were calculated using a traditional heuristic search (100 replicates of TBR each bootstrap replicate, 10 trees saved per TBR) with 1000 bootstrap replications. Bremer supports were calculated by running the script “Bremer.run” and checking this using heuristic searches saving suboptimal trees up to 8 steps longer and running “Bremer supports” in TNT, then repeating with different random seeds 10 times.

### Nomenclatural Acts

The electronic edition of this article conforms to the requirements of the amended International Code of Zoological Nomenclature, and hence the new names contained herein are available under that Code from the electronic edition of this article. This published work and the nomenclatural acts it contains have been registered in ZooBank, the online registration system for the ICZN. The ZooBank LSIDs (Life Science Identifiers) can be resolved and the associated information viewed through any standard web browser by appending the LSID to the prefix "http://zoobank.org/". The LSID for this publication is: urn:lsid:zoobank.org:pub:AA4CAF05-484C-40E7-995D-9DE61D488111. The electronic edition of this work was published in a journal with an ISSN (1932–6203), and has been archived and is available from the following digital repositories: PubMed Central (http://www.ncbi.nlm.nih.gov/pmc), LOCKSS (http://www.lockss.org).

## Results

### Systematic Paleontology

Dinosauria Owen, 1842 [23]

Ornithischia Seeley, 1887 [24]

Ceratopsia Marsh, 1890 [25]

Chaoyangsauridae Zhao et al., 2006 [3]

#### Type Genus

*Chaoyangsaurus* Zhao et al., 1999 [2]

#### Definition

A stem-based taxon defined as all ceratopsians more closely related to *Chaoyangsaurus youngi* than to *Psittacosaurus mongoliensis* [26] or *Triceratops horridus* [27].

#### Revised Diagnosis

Chaoyangsaurids may be distinguished from other ceratopsians by the following synapomorphies: semicircular ventral process near the medial face of the mandibular glenoid [3], expanded, flat dorsal surface of the squamosal with a stalked quadrate process, deep sulcus dividing the quadrate condyles, ventral margin of the angular extending laterally to form a ridge with a distinct concavity formed above the ridge, predentary reduced and much shorter than premaxillary oral margin, dorsal and ventral margin of the dentary converged rostrally more than 20% of the depth.

*Hualianceratops* gen.nov.

urn:lsid:zoobank.org:act:D96319BA-6380-47D6-9512-5BDA15221A00

Type Species.

*Hualianceratops wucaiwanensis* gen. et sp. nov.

urn:lsid:zoobank.org:act:DEEB3095-CB69-47CD-91FC-2D01D9F429D5

#### Etymology

“Hualian” means ornamental face, referring to the texture found on most part of the skull, combined with *ceratops* (horned face) from the Greek, a common suffix for horned dinosaurs; “wucaiwan” (Chinese: five color bay) for the area where the specimen was discovered.

#### Holotype

IVPP V18641, articulated right maxilla, jugal, postorbital and partial squamosal, articulated right quadrate and partial quadratojugal, articulated left partial jugal, quadratojugal and quadrate, left partial squamosal, most of the mandible, and postcranial fragments including a nearly complete left pes (Figs 1–9; also see S1 File).

**Fig 1:**
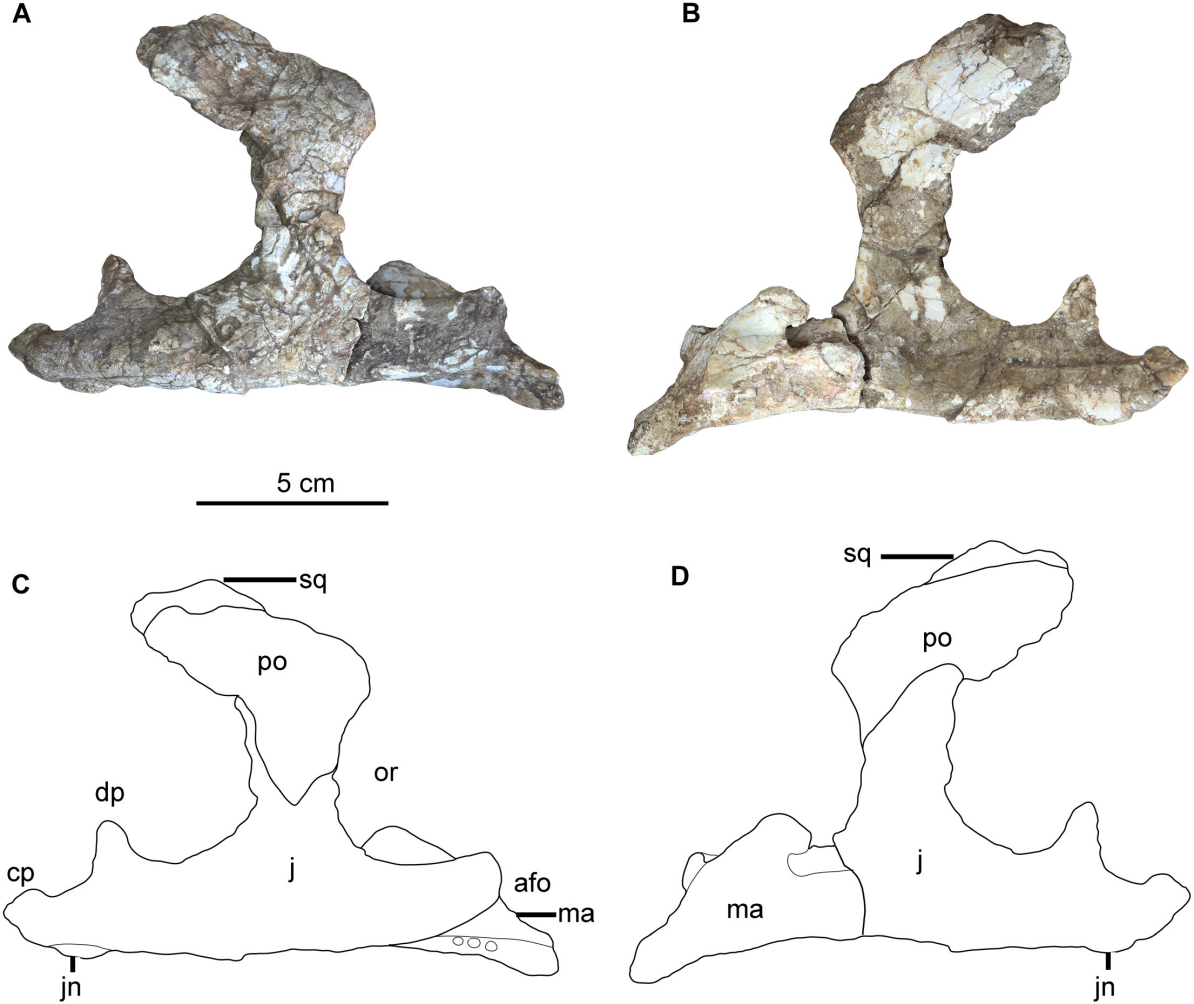
The articulated right maxilla and jugal of *Hualianceratops wucaiwanensis* (IVPP V18641). (A) photograph in lateral view, (B) photograph in medial view, (C) drawing in lateral view, (D) drawing in medial view. Abbreviations: afo, antorbital fossa; cp, caudomedial process of the jugal; dp, caudodorsal process of the jugal; j, jugal; jn, jugal node; ma, maxilla; or, orbit; po, postorbital; sq, squamosal.

**Fig 2:**
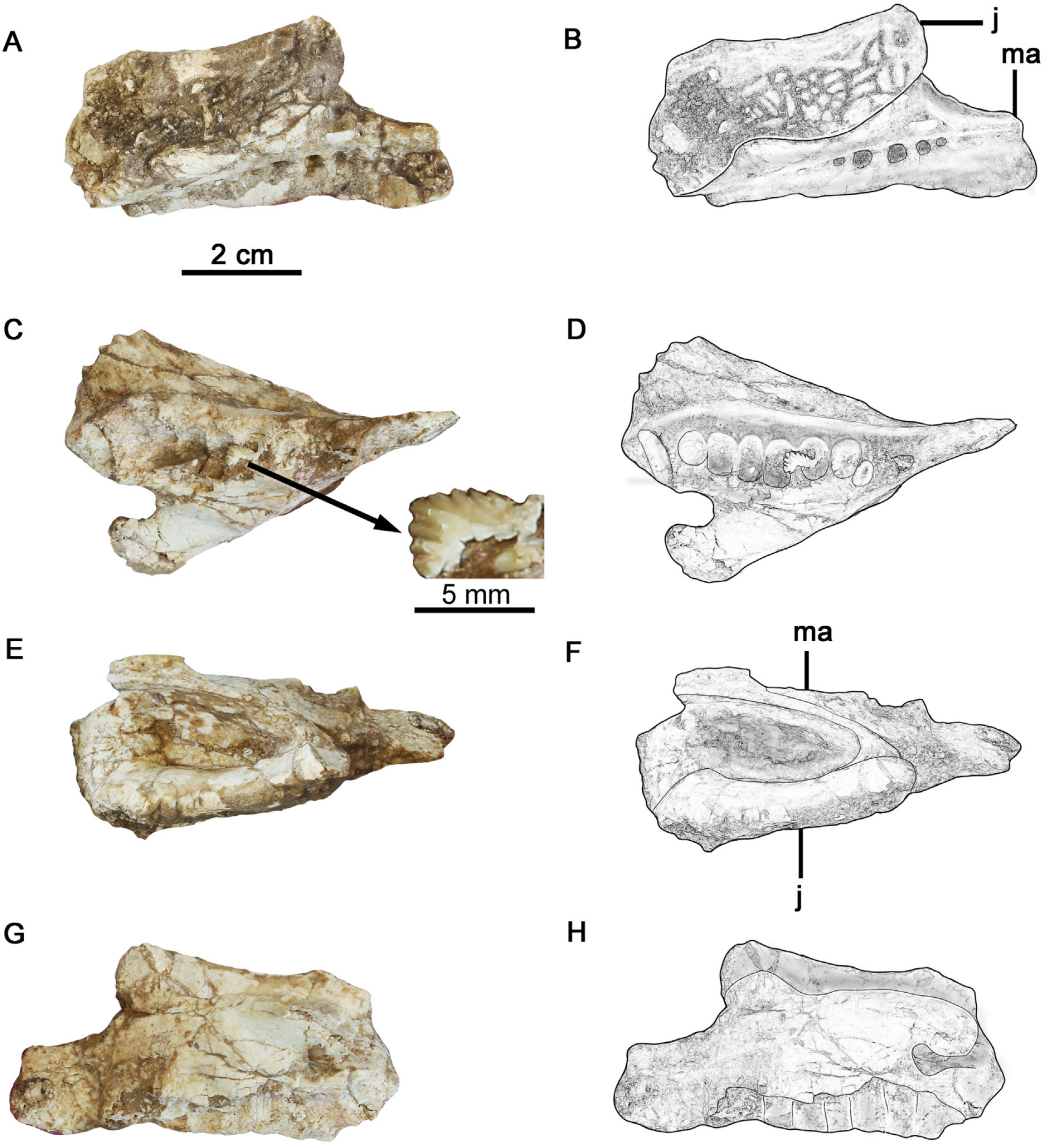
The articulated right maxilla and partial jugal of *Hualianceratops wucaiwanensis* (IVPP V18641), the broken rostral part of the elements in Fig 1. (A) photograph in lateral view, (B) drawing in lateral view, (C) photograph in ventral view with inset of a tooth enlarged, (D) drawing in ventral view, (E) photograph in dorsal view, (F) drawing in dorsal view, (G) photograph in medial view, (H) drawing in medial view. Abbreviations: ma, maxilla; j, jugal.

**Fig 3:**
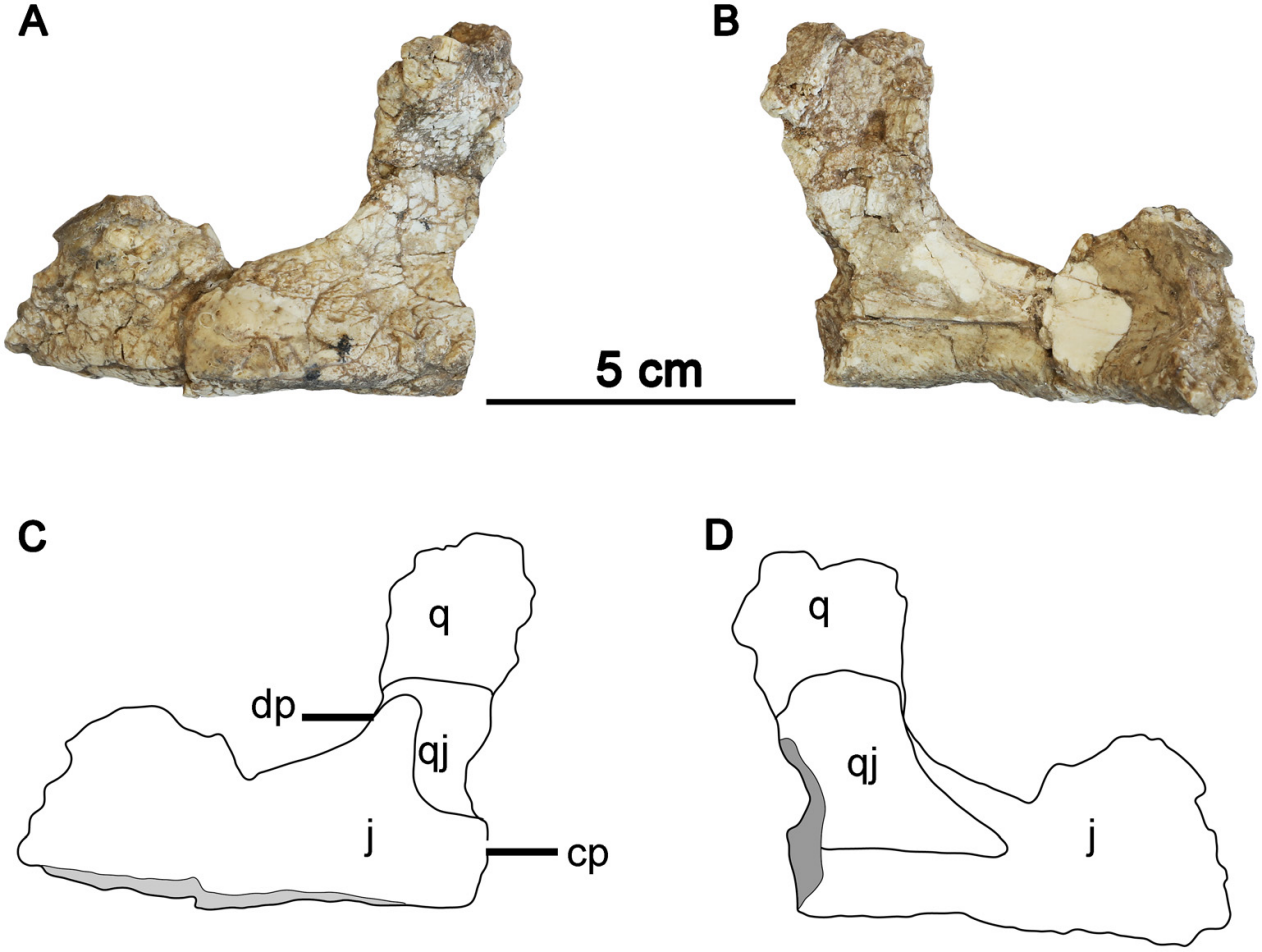
The articulated left partial jugal and quadratojugal of *Hualianceratops wucaiwanensis* (IVPP V18641). (A) photograph in lateral view, (B) photograph in medial view, (C) drawing in lateral view; (D) drawing in medial view. Abbreviations: cp, caudomedial process of the jugal; dp, caudodorsal process of the jugal; qj, quadratojugal; q, quadrate; j, jugal.

**Fig 4:**
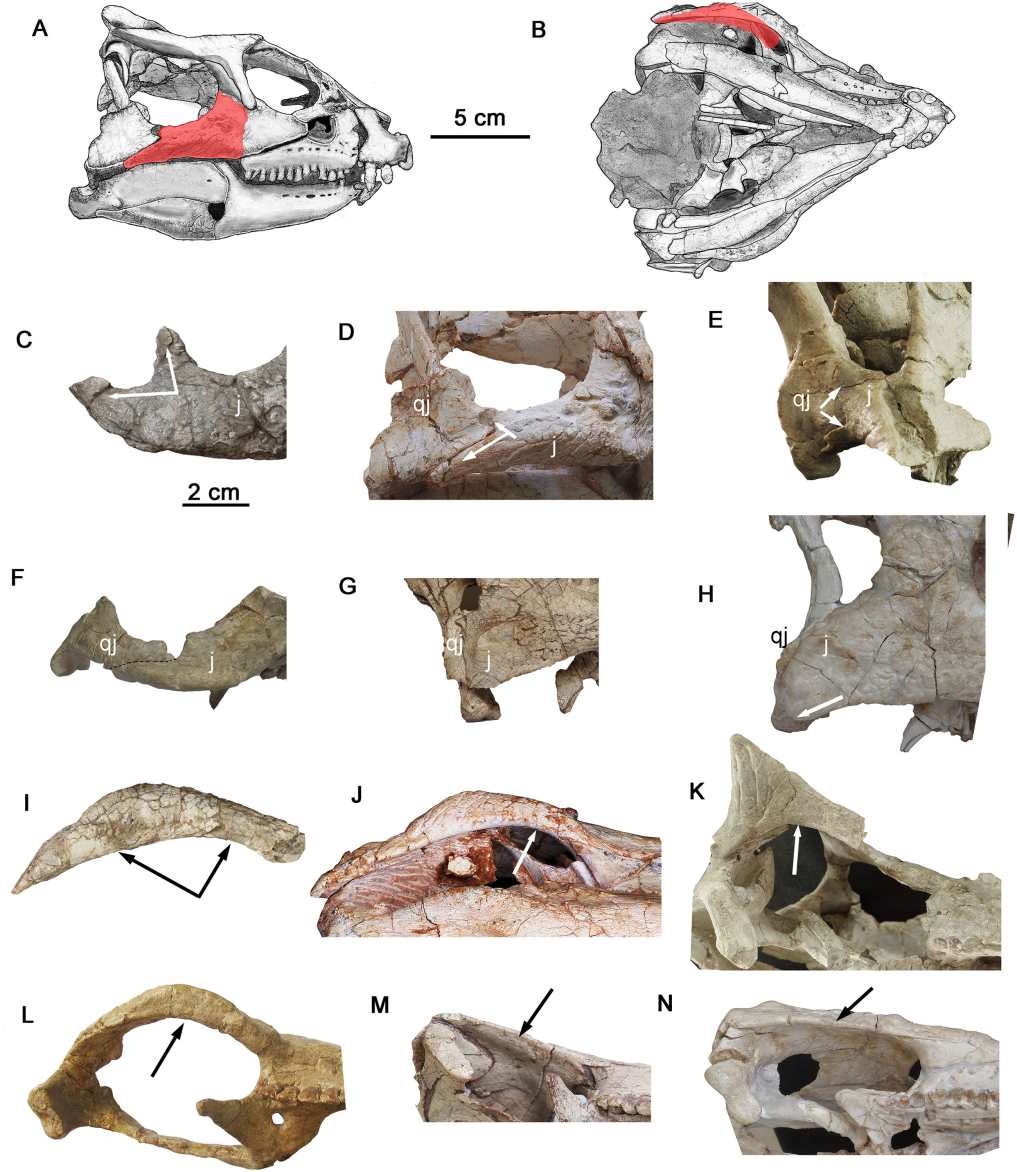
Comparisons of the infratemporal ramus of the jugal in basal ceratopsians. Outline drawing of the holotype skull of *Yinlong downsi* showing the infratemporal ramus of the jugal (red) in lateral view (A) and ventral view (B), and the infratemporal ramus in basal ceratopsians in right lateral view (C-H) and ventral view (I-N). (C) *Hualianceratops wucaiwanensis* (IVPP V18641), (D) *Yinlong downsi* (IVPP V14530), (E) *Hongshanosaurus houi* (IVPP V12617), (F) *Chaoyangsaurus youngi* (IGCAGS V371), (G) *Liaoceratops yanzigouensis* (IVPP V12633), (H) *Archaeoceratops oshimai* (IVPP V11114). (I) *Hualianceratops wucaiwanensis* (IVPP V18641), (J) *Yinlong downsi* (IVPP V14530), (K) *Hongshanosaurus houi* (IVPP V12617), (L) *Chaoyangsaurus youngi* (IGCAGS V371), (M) *Liaoceratops yanzigouensis* (IVPP V12633), (N) *Archaeoceratops oshimai* (IVPP V11114). The arrows show the caudal process of the infratemporal ramus of the jugal. Abbreviations: j, jugal; qj, quadratojugal.

**Fig 5:**
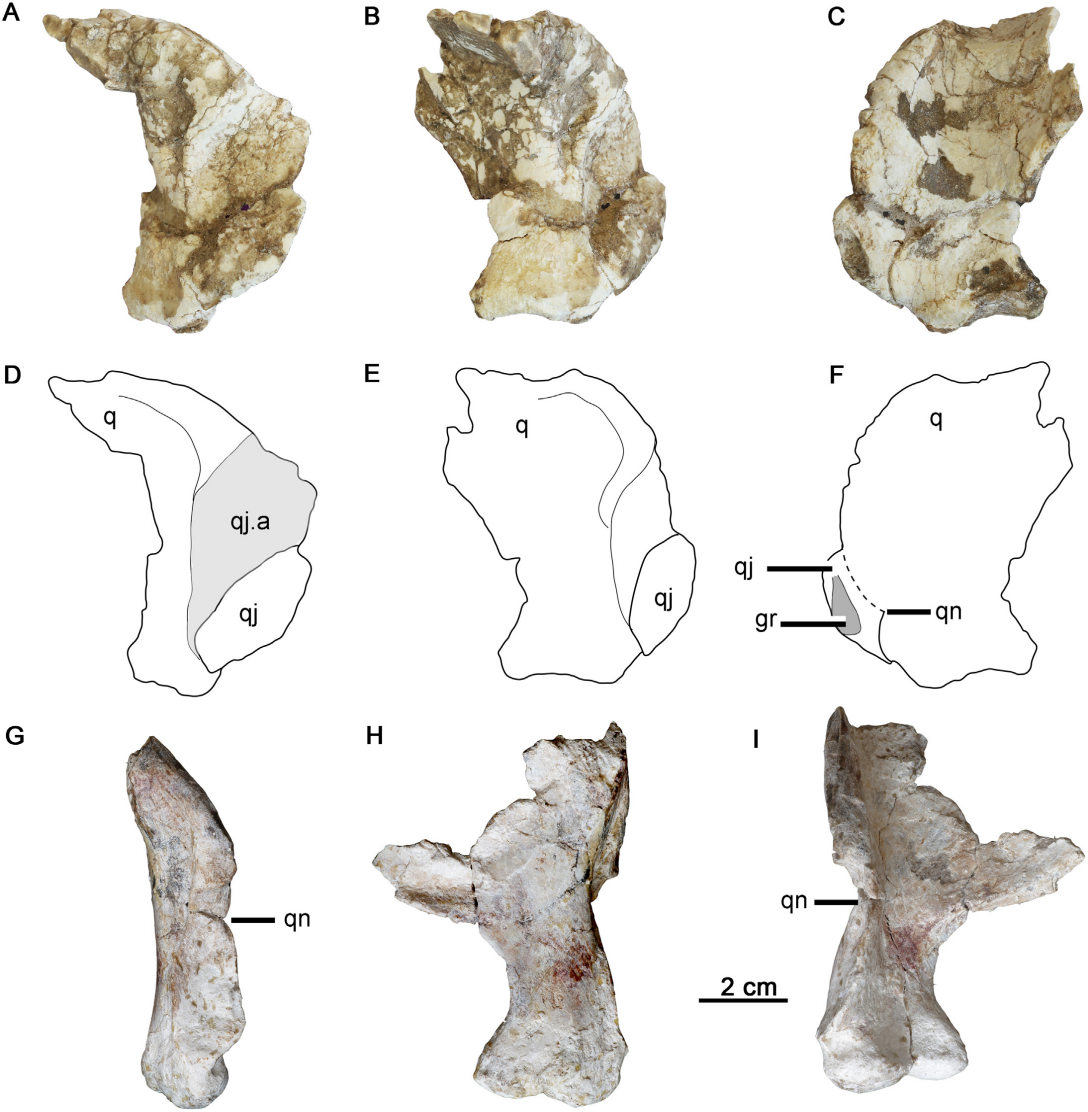
Comparison of the quadrates in *Hualianceratops wucaiwanensis* and *Yinlong downsi*. (A-F) right quadrate and quadratojugal of *Hualianceratops wucaiwanensis*. (A) photograph in lateral view, (B) photograph in caudal view, (C) photograph in rostral view, (D) drawing in lateral view, (E) drawing in caudal view, (F) drawing in rostral view. (G-I) left quadrate of *Yinlong* (IVPP V18637) flipped for comparison with the right side. (G) Photograph in lateral view, (H) photograph in caudal view, (I) photograph in rostral view. Abbreviations: gr, groove; q, quadrate; qj, quadratojugal; qj.a, articulation with the quadratojugal; qn, quadrate notch.

**Fig 6:**
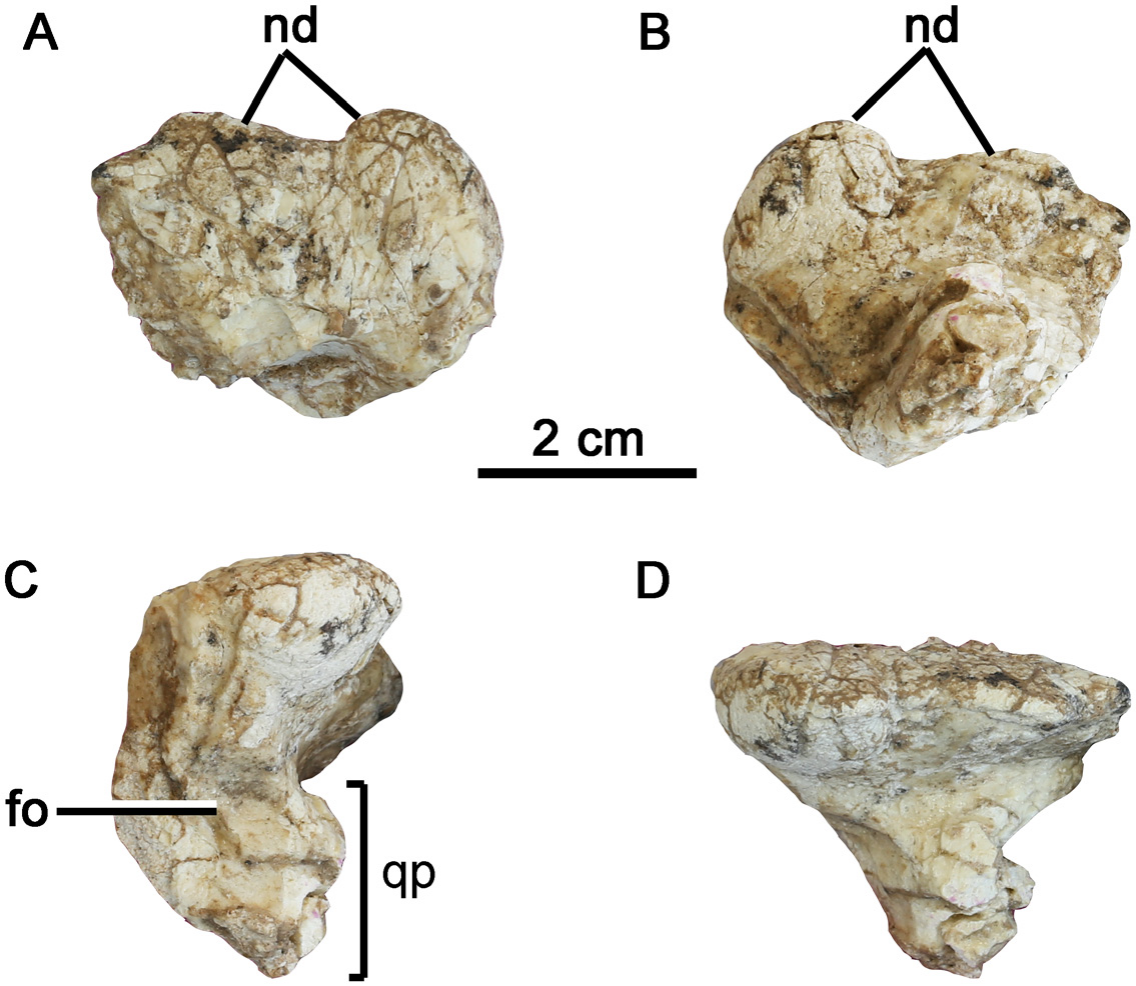
The squamosal of *Yinlong downsi* (IVPP V18641). (A) dorsal view, (B) ventral view, (C) left lateral view, (D) caudal view. Abbreviations: fo, fossa on the lateral surface; nd, nodes on the caudal margin; qp, stalked quadrate process.

**Fig 7:**
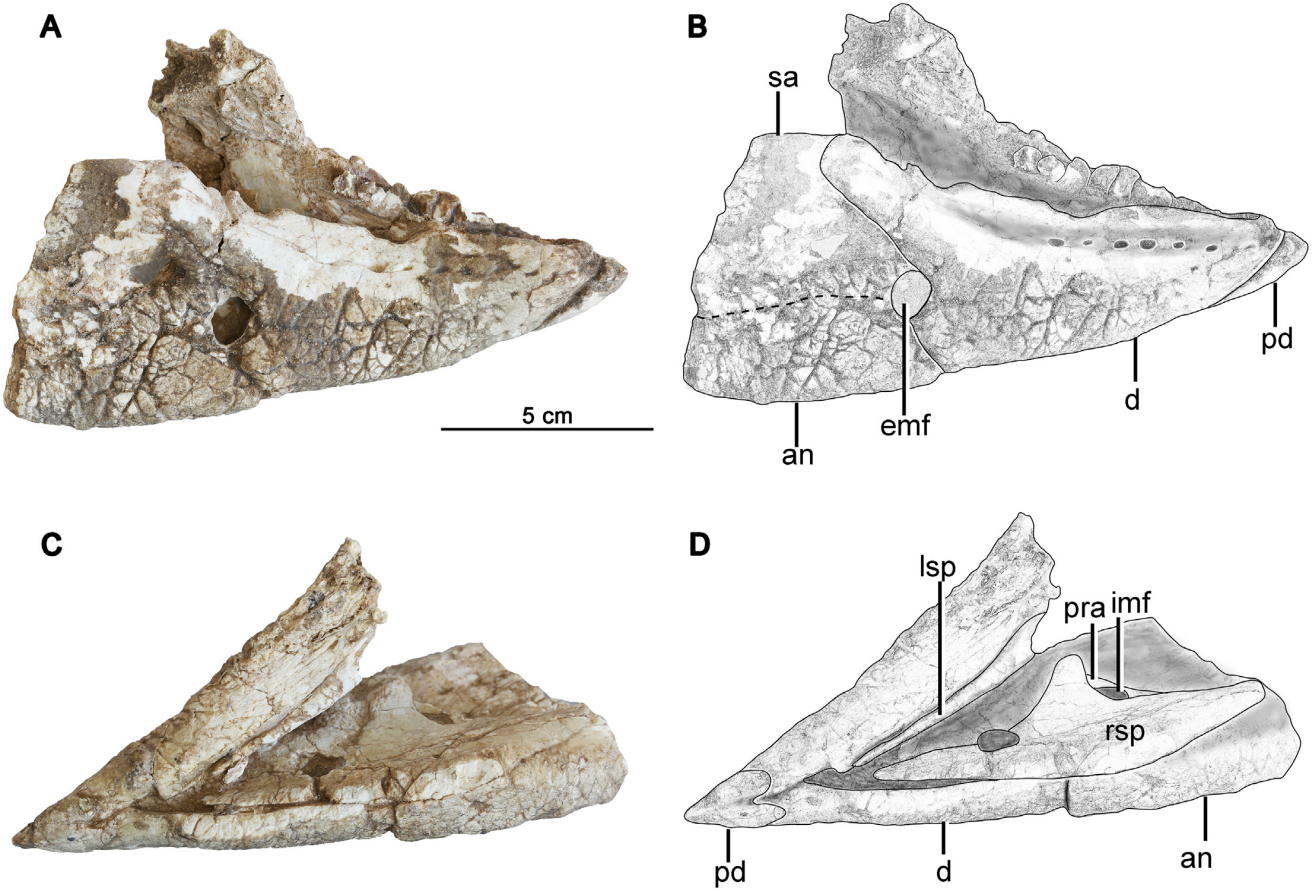
The mandible of *Hualianceratops wucaiwanensis* (IVPP V18641). (A) photograph in lateral view, (B) drawing in lateral view, (C) photograph in ventral view, (D) drawing in ventral view. Abbreviations: an, angular; d, dentary; emf, external mandibular fenestra; imf, inner mandibular fenestra; lsp, left splenial; pra, prearticular; pd, predentary; rsp, right splenial; sa, surangular.

**Fig 8:**
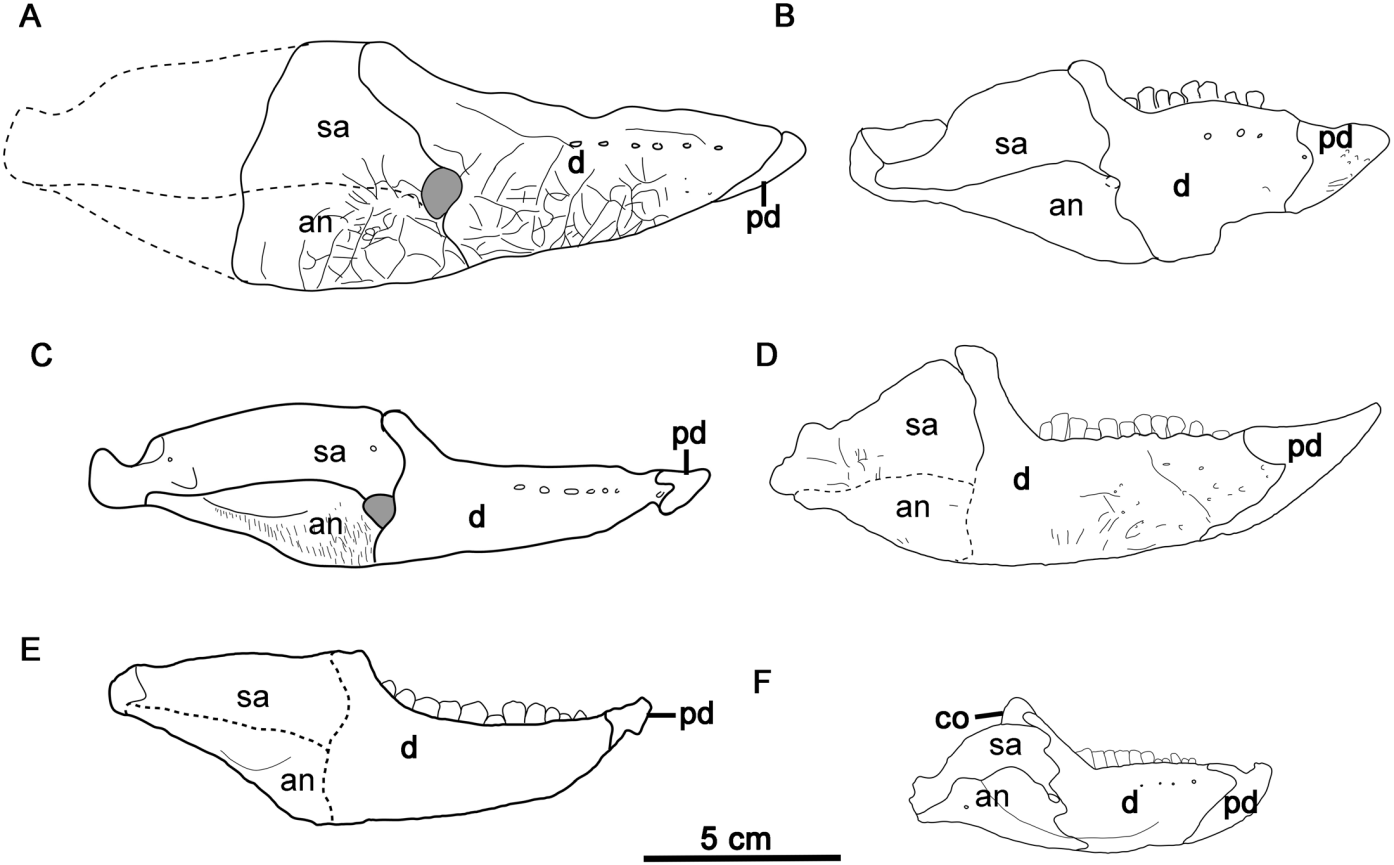
Comparison of the mandible in basal ceratopsians in right lateral view. (A) *Hualianceratops wucaiwanensis* (IVPP V18641), (B) *Hongshanosaurus houi* (IVPP V12617), (C) *Yinlong downsi* (IVPP V14530), (D) *Archaeoceratops oshimai* (IVPP V11114), (E) *Chaoyangsaurus youngi* (IGCAGS V371), (F) *Liaoceratops yanzigouensis* (IVPP V12633). Abbreviations: an, angular; co, coronoid; d, dentary; pd, predentary; sa, surangular.

**Fig 9:**
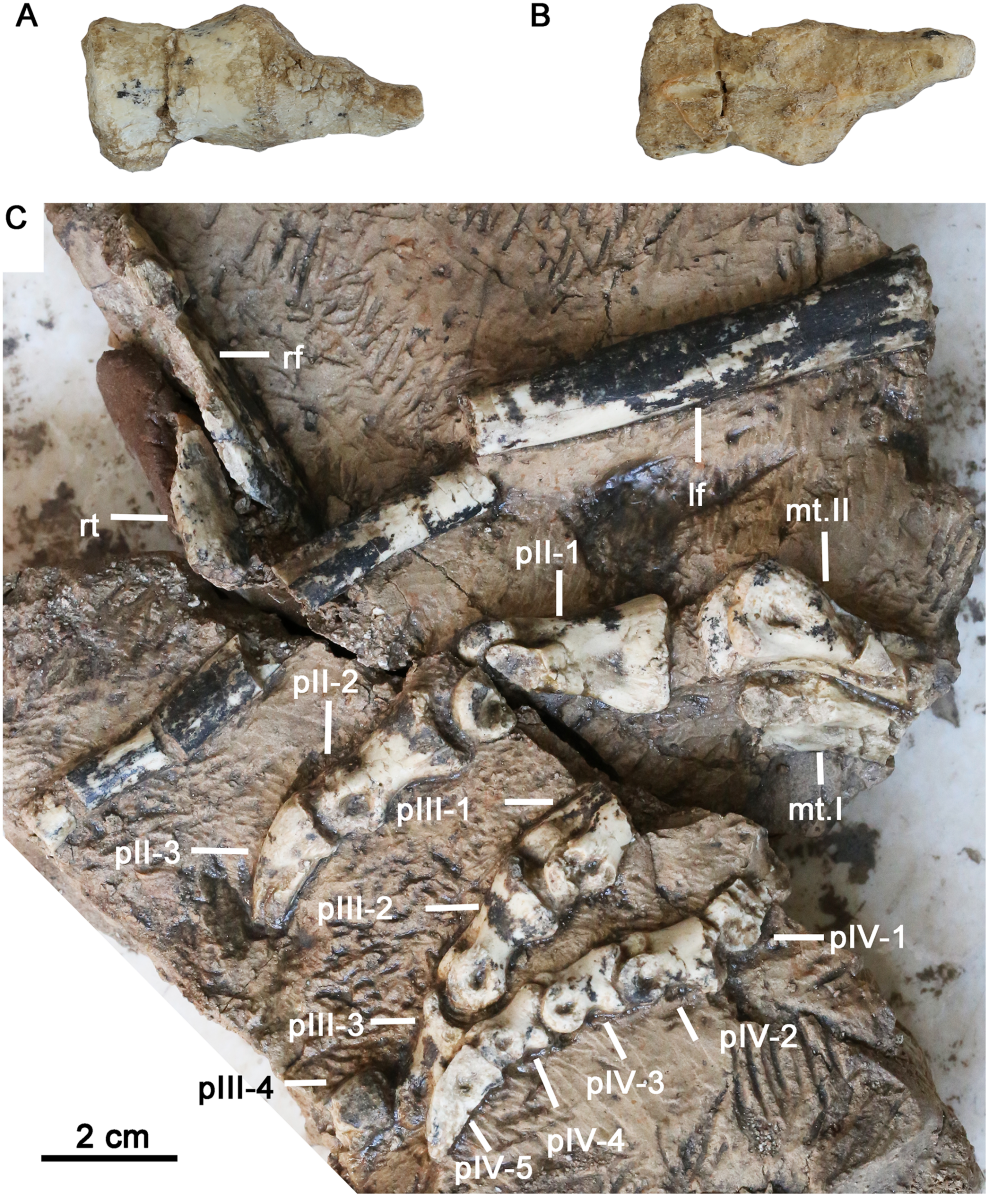
Postcranial material of *Hualianceratops wucaiwanensis* (IVPP V18641). (A-B) two fused sacral vertebrates, cranial end is to the left. (A) ventral view, (B) dorsal view. (C) partial hind limb and left pes of *Hualianceratops wucaiwanensis*. Abbreviations: rf, right fibula; rt, right tibia; lf, left fibula; mt.I-II, metatarsal I-II; pII-IV, pes digits II-IV.

#### Locality and Horizon

Wucaiwan locality, Junggar Basin, Xinjiang, China; upper part of Shishugou Formation, correlated with the early Late Jurassic (Oxfordian). The holotype was found in fluvial mudstones in the same part of the Shishugou Formation as *Yinlong*, between “big white” and tuff 4 [14, 28], 159.7+/-0.3 and 162.2+/-0.2 Ma.

#### Diagnosis

A basal ceratopsian distinguished from other chaoyangsaurids by five autapomorphies: a prominent caudodorsal process on the infratemporal ramus of the jugal, a robust quadrate with an expansive quadratojugal facet, a transversely expanded rostral margin of the quadrate above the quadratojugal facet, a prominent notch near the ventral region of the quadrate, and a deep and short dentary. Unique combination of character states includes: strongly rugose sculpturing present on the lateral surface of the dentary (distinct from *Chaoyangsaurus* and *Yinlong*, but present in *Xuanhuaceratops*), transversely expanded ventral surface of the infratemporal ramus of the jugal (distinct from *Yinlong*, but present in *Chaoyangsaurus*), ventral grooves present on the quadratojugal for articulating with the caudal end of the jugal (distinct from *Xuanhuaceratops*, but present in *Chaoyangsaurus* and *Yinlong*).

## Description and Comparisons

### Skull

The skull is estimated to be 25 cm long (from the rostral end to the quadrate condyles) based on the preserved materials. It is significantly larger and more robust than the *Yinlong downsi* holotype (IVPP V14530), which measures 18 cm long, and slightly smaller than the largest *Yinlong* skull (IVPP V18637) based on the jugal, quadrate and dentary (Table 1). The preorbital region is missing, but the ventral margin of a large antorbital fossa is preserved. The orbit is circular and seems to be the largest opening of the skull. The infratemporal fenestra is deep and subelliptical in outline, and slightly narrower than the orbit. The jugal-postorbital bar is wide compared to that of *Psittacosaurus* [29], but still relatively narrower than that of other neoceratopsians, such as *Liaoceratops* [4]. The textured ornamentation is well developed and present in most of the preserved skull bones, including the jugal, quadratojugal, postorbital squamosal, dentary, surangular, and angular. It is similar in development to that of *Xuanhuaceratops* [3], and more developed than in other basal ceratopsians. Textured ornamentation is usually present on the sub-temporal ramus of the jugal, postorbital, and angular in chaoyangsaurids and the basal neoceratopsians *Liaoceratops*, *Archaeoceratops*, and *Auroraceratops*, but is absent in *Aquilops* and more derived neoceratopsians [9]. These ornamentations are usually textured and grooved, and differ from the nodular-like ornamentation of pachycephalosaurs [30].

**Table 1:** Skull measurements of *Hualianceratops* (IVPP 18641) and *Yinlong downsi* [IVPP 14530 (holotype), IVPP 18637], in millimeters.

Elements	Variable	IVPP V18641	IVPP V14530	IVPP V18637
Jugal	total length	133.8	108.8	140*
	length of the orbital ramus	46.9	33.6	––
	depth at the base of the orbital ramus	30.0	27.5	––
	length of the infratemporal ramus	69.2	65.7	––
	depth at the base of the infratemporal ramus	32.1	25.5	––
	length from the distal end of the postorbital ramus to its base	72.8	52.9	75.8
	thickness of the ventral margin near the base of the infratemporal ramus	19.4	12.4	––
quadrate	length from the quadrate head to the ventral margin	71.9	57*	86.5
	width of the quadrate condyles	30.9	25.4	32.8
	thickness of the ventral region	14	––	15.7
	maximum width in lateral view	36.5	17.7	21.2
dentary	length along the ventral margin	83.2	81.6	––
	depth of the rostral end	26.7	19.8	––
	depth at caudal end of toothrow	43.3	26.2	––
	maximum depth	63.3	40.4	––

*estimated.

### Maxilla

The right maxilla is missing its rostral region as well as part of its dorsal and caudal margins (Figs 1 and 2). Most of the tooth-bearing portion is preserved although most crowns are missing; at least nine alveoli are preserved (Fig 2). The antorbital fossa is partially preserved.

The toothrow is gently bowed medially, as in all ceratopsians and most ornithischians. The maxilla is rostrocaudally elongate, and its caudal end is overlapped almost entirely by the infraorbital ramus of the jugal. The toothrow is medially inset to form a deep buccal emargination, which is deeper than that of *Yinlong* [1, 14]. The emargination is well-defined caudally, but quickly becomes less distinct rostrally. At least six large, subcircular nutrient foramina form a line along the lateral surface of the maxilla, parallel to the alveolar margin and immediately below the buccal emargination.

The dorsal margin of the preserved maxilla is smooth and slightly curved where it forms the well-defined ventral edge of the large antorbital fossa. The antorbital fossa has a relatively straight caudal margin (formed by the jugal) and a rostroventrally sloping ventral margin. The shape of the complete antorbital fossa cannot be determined. The ventral edge of the antorbital fossa is significantly lower than that of the orbit, as in other ceratopsians. Caudally, the maxilla tapers and is broadly overlapped by the ventromedial surface of the infraorbital ramus of the jugal. The medial surface of the maxilla is rugose and flattened, and seems to have been compressed during preservation. The articulations with the lacrimal, premaxilla, and nasal are not preserved. In overall morphology, the preserved portion of the maxilla is similar to that of *Yinlong*.

### Jugal

The right jugal and the caudal portion of the left jugal are well preserved (Figs 1 and 3). The infraorbital ramus of the right jugal is separated from the main body and attached to the maxilla (Fig 2). The depth of the suborbital and subtemporal rami are relatively equal, as in most basal ceratopsians; the infratemporal ramus is substantially deeper in more derived neoceratopsians (e.g., *Liaoceratops* [4], *Yamaceratops* [8]). The infraorbital ramus is much shorter than the infratemporal ramus, as in *Psittacosaurus* and derived neoceratopsians such as *Protoceratops*, but in contrast to the basal neoceratopsians *Liaoceratops*, *Archaeoceratops* and *Yamaceratops* which have relatively shorter infratemporal rami [4, 5, 8]. The lateral surface of the entire jugal is rugose and strongly textured, as in *Xuanhuaceratops* [3], *Archaeoceratops* [5] and *Auroraceratops* [11]. In *Yinlong*, the textured surface is restricted to the postorbital and infratemporal rami and absent on the infraorbital ramus [14]. This feature is absent or weak in *Chaoyangsaurus* [2], *Psittacosaurus* [29], *Liaoceratops* [4] and *Aquilops* [9]. Large oval nodules are present on the jugal in *Yinlong* [14], but are absent in *Hualianceratops*.

The infraorbital ramus is transversely thick, tapers rostrally, and forms the entire caudal margin of the antorbital fossa. The dorsal margin of the ramus is smooth and curved rostrodorsally where it forms the ventral margin of the orbit. The body of the infraorbital ramus thins mediolaterally to the narrow orbital margin. The medial surface of the infraorbital ramus is not exposed.

The tall postorbital ramus of the jugal is rostrocaudally wide and contributes to a wide jugal-postorbital bar, as in basal neoceratopsians (e.g., *Liaoceratops* [4]; *Archaeoceratops* [5]). In *Psittacosaurus* the jugal-postorbital bar is much narrower [31]. In medial view, the distal end of the postorbital process of the jugal tapers caudodorsally, reaching to the medioventral surface of the main body of the postorbital. In lateral view, it is broadly overlapped by the jugal process of the postorbital so that most of the postorbital ramus of the jugal is concealed, as in *Yinlong* and *Psittacosaurus* [31], but unlike the situation in *Liaoceratops* and *Archaeoceratops* where the exposed jugal occupies the rostral half of the jugal-postorbital bar [4, 5].

The robust infratemporal ramus is strongly arched laterally like that of *Yinlong*, *Chaoyangsaurus* and *Psittacosaurus*, whereas it is nearly straight or just slightly curved in basal neoceratopsians, such as *Liaoceratops*, *Archaeoceratops* and *Auroraceratops* [4, 5, 11] (Fig 4). It has a subtriangular cross section with a thin dorsal edge and thickened ventral edge (Figs 1 and 3). The surface of the ventral margin is textured and flattened in both the jugals. This is similar to the situation in *Chaoyangsaurus* (IGCAGS V371) and *Psittacosaurus* (e.g., IVPP V12617), but unlike *Yinlong* (e.g., IVPP V14530) and other basal neoceratopsians which have thin ventral edges on their jugal infratemporal rami. There is no trace of an epijugal or jugal horn, as in *Yinlong* and *Chaoyangsaurus*.[32].

The caudal end of the infratemporal ramus bifurcates into a caudomedial process and a caudodorsal process (Figs 1 and 3). The larger caudomedial process tapers and curves medially, reflecting the lateral arching of the entire ramus. The dorsal margin of its distal end is thin and fits into a deep sulcus on the lateral surface of the quadratojugal, as in *Yinlong* [14]. The smaller caudodorsal process extends nearly directly dorsally from the infratemporal ramus. It is transversely compressed, and triangular in lateral view (Figs 1 and 3); its rostral margin forms the caudoventral margin of the infratemporal fenestra. The caudodorsal process fits onto a facet on the lateral surface of the rostral quadratojugal, nearly eliminating the quadratojugal from the margin of the infratemporal fenestra. This differs from the jugal of *Yinlong*, where the caudodorsal process is relatively smaller and extends caudally along the inferior margin of the infratemporal fenestra, allowing the quadratojugal a larger contribution to the infratemporal fenestra margin [1]. In *Psittacosaurus*, the caudodorsal process is nearly as long as the caudomedial process, but as in *Yinlong* extends caudally and does not curve dorsally along the rear of the infratemporal fenestra (Fig 4C). In most neoceratopsians the caudodorsal process is lost (e.g., *Liaoceratops*) (Fig 4). A prominent node is present on the ventral surface of the left infratemporal ramus of *Hualianceratops*, but is absent from the right jugal (Figs 1 and 3); this feature may vary per individual.

### Quadratojugal

Both of the quadratojugals are partially preserved (Figs 3 and 5). The rostral part of the left quadratojugal is preserved and articulated with the jugal and quadrate (Fig 3). The caudoventral part of the right quadratojugal is also preserved and articulated with the quadrate (Fig 5). The preserved quadratojugal is mediolaterally compressed and is ornamented on its exposed lateral surface.

In lateral view, the rostral margin of the quadratojugal follows the curve of the infratemporal fenestra, but is nearly completely covered laterally by the jugal. Only a small sliver of rostrodorsal quadratojugal was apparently exposed along the margin of the infratemporal fenestra, although poor preservation of the associated quadrate makes this difficult to confirm. Dorsally, the quadratojugal appears to extend onto the rostromedial surface of the quadrate. However, this area is poorly preserved and the quadratojugal is broken along its dorsal margin. The quadratojugal extended distally to reach the lateral quadrate condyles.

The rostral quadratojugal is incised by a deep sulcus for articulation with the caudomedial process of the infratemporal ramus of the jugal, as in *Yinlong* (Fig 5C and 5F). This feature is very weakly developed in *Chaoyangsaurus* and *Xuanhuaceratops* (pers. obs.), and absent in other ceratopsians. The quadratojugal tapers rostrally and extends forward to reach the main body of the jugal, deep to its infratemporal ramus (Fig 3D). The straight ventral margin is thin and oriented horizontally, while the rostrodorsal margin curves along the caudoventral margin of the infratemporal fenestra. The caudoventral margin of the quadratojugal is indented by a large, oval fossa or groove that faces rostrolaterally (Fig 5C). This fossa is adjacent to a large notch in the lateral margin of the quadratojugal wing of the quadrate.

### Quadrate

The right quadrate is well preserved, but is missing most of the pterygoid wing (Fig 5). The shaft is robust and rostrocaudally wide. The dorsal portion of the shaft is strongly deflected caudodorsally (Fig 5A), as in *Psittacosaurus* [33], but unlike that of *Yinlong* and other neoceratopsians which have relative straight to slightly curved proximal quadrate shafts [1]. Because only one quadrate is preserved, the possibility that the strong deflection of the quadrate head may be accentuated by or due to distortion cannot be eliminated. The shaft becomes rostrocaudally narrower but maintains a robust rostral margin (about 10.5 mm in transversely width) above the quadratojugal wing as it curves caudally to approach the quadrate head, as in *Psittacosaurus* (IVPP V12617). This condition differs from that of *Yinlong* and other basal neoceratopsians which have transversely narrow rostral margins [14]. The pterygoid wing extends rostromedially from the shaft as a deep, thin plate that is broken near its base.

Distal to the thickened rostrodorsal margin of the shaft, the broad quadratojugal wing supports an enormous quadratojugal facet whose width is 57% its length (27.8 mm in maximum width, 49 mm in length). This facet occupies half the length of the quadrate shaft, as in the largest individual of *Yinlong* (IVPP V18637). However, the latter has a much narrower facet (38% width to length; 15 mm in maximum width, 40 mm in length) (Fig 5G). In other known neoceratopsians and *Psittacosaurus*, the quadratojugal facet occupies less than half the length of the distal quadrate shaft [33]. This indicates an extensive overlap between the quadrate and quadratojugal in *Hualianceratops*. The caudal margin of the quadratojugal facet is distinct and raised relative to the quadrate shaft. The facet occupies nearly the entire width of the lateral margin of the quadrate shaft, and extends distally nearly to the condyles.

A small, oval foramen pierces through the quadrate shaft caudal to the quadratojugal facet, as in *Yinlong* and basal neoceratopsians (e.g., *Auroraceratops* [11]; *Liaoceratops*, IVPP V12738). Ventrally, the quadrate expands slightly forming the lateral and medial condyles. The medial condyle is just slightly smaller but extends more ventrally than the lateral condyle. The condyles are compressed rostrocaudally and separated by a broad, shallow sulcus. This is different from *Yinlong* (IVPP V18637), *Chaoyangsaurus* (IGCAGS V371), and *Xuanhuaceratops* [3], where the condyles are more rounded and prominent and separated by a deep and narrow intercondylar sulcus (Fig 5H and 5I). In this respect, the condyles of *Hualianceratops* are more similar to those of other neoceratopsians (e.g., *Liaoceratops*) and *Psittacosaurus* (e.g., IVPP V12617).

A prominent V-shaped notch incises the ventral region of the quadratojugal wing of the quadrate (Fig 5C and 5F). This notch is covered laterally by the quadratojugal, but is visible in medial view. A quadrate notch is present in *Yinlong* (e.g., IVPP V18637) (Fig 5G), but is shallow, incises the rostral margin of the quadratojugal facet, and is positioned slightly above the midlength of the quadratojugal wing (Fig 5G). Quadrate notches (also referred to a paraquadratic notches) such as that found in *Hualianceratops* and *Yinlong* are widespread in ornithopods, but are absent/weak or unknown in other ceratopsians [19]. The notch shape in *Hualianceratops* appears to be unique among ornithischians.

### Postorbital

The right postorbital is partially preserved in articulation with the jugal and the postorbital ramus of the squamosal (Fig 1). The articulation with the frontal and parietal are missing. The main body of the postorbital was compressed and flattened mediolaterally during preservation. Its lateral surface retains the same rugose texturing as found on the jugal and quadratojugal.

The jugal process of the postorbital is mediolaterally compressed and tapers rostroventrally. It broadly overlaps a facet on the rostrolateral surface of the postorbital process of the jugal, and forms about half of the caudal margin of the orbit and the rostral half of the broad postorbital-jugal bar.

The caudal margin of the jugal process of the postorbital grades smoothly into the ventral margin of the squamosal process. The squamosal process expands medially into a subtriangular cross section, and tapers caudally. The tapered rostral end of the postorbital process of the squamosal extends nearly to the rostral limit of the infratemporal fenestra and appears to articulate with the dorsomedial aspect of the squamosal process of the postorbital. The squamosal process of the postorbital appears to be relatively smaller than that of *Yinlong*, suggesting a relatively smaller infratemporal fenestra. The postorbital appears to have formed a portion of the rostrolateral margin of the supratemporal fenestra. The caudal and lateral margins of the postorbital are eroded, and no nodes or nodules can be detected.

### Squamosal

The left squamosal is partially preserved (Figs 1 and 6), and is very similar to that of *Yinlong* in having a flat, expanded dorsum and a constricted, stalked quadrate process. The dorsal surface is flattened and textured, and two large nodes are present along the caudal margin, as in *Yinlong*. These thick nodes become somewhat dorsoventrally compressed towards the margin of the element. The lateral node occurs at the caudolateral corner of the squamosal and is more robust than the more medial one.

The lateral node extends laterally to overhang the quadrate process at an angle of 120 degrees, whereas the caudal node extends caudodorsally at a right angle to the quadrate process.

Ventrally, the squamosal contracts to form a discrete, stalk-like quadrate process. The cotylus and cotylar processes are broken and absent. In lateral view, a fossa is present on the lateral surface of the quadrate process that is confluent with the caudodorsal margin of the lower temporal fenestra, as occurs in many ornithischians (Fig 6C).

### Mandible

The paired mandibles are partially preserved in articulation but are missing most of the postdentary series (Fig 7). The right dentary is compressed and mediodorsally distorted; the left dentary is straight in lateral view although the surface is seriously damaged.

#### Predentary

The predentary is partially preserved. It is triangular in ventral view, and converges to a narrow rostral margin; it appears to have had a rounded rather than a sharp ventral keel. The caudoventral process is well-preserved and covers most of the mandibular symphysis, although the caudalmost symphysis remains exposed. Caudally the process bifurcates, as in other basal ceratopsians (e.g., *Liaoceratops* [4]; *Psittacosaurus*, IVPP V12617), and most ornithopods (e.g., *Haya* [34] and *Changchunsaurus* [35]) (Fig 7C). The condition in *Yinlong* is not known. Both dorsolateral processes are missing. The tip of the predentary is broken below the level of the dorsal margin of the dentary.

#### Dentary

Both dentaries are preserved. The right one is nearly complete but its oral margin is damaged (Fig 7). Although the entire toothrow is preserved, an accurate tooth count is not possible due to remaining matrix, but at least seven teeth are preserved in the left dentary. The first tooth lies immediately behind the predentary, precluding a diastema.

The dentary is deep and short, and its dorsal and ventral margins converge rostrally. It measures 83.2 mm in length along its ventral margin, and has depths of 26.7 mm (32% length) at the rostral end, 43.3 mm (52% length) at the rear of the toothrow, and 63.3 mm (76% length) at the apex of the coronoid process. In the holotype of *Yinlong* (IVPP V14530), the dentary has a length of 81.6 mm at the base, just slightly shorter than that of *Hualianceratops* (Fig 8A and 8C). However, the *Yinlong* holotype has depths of 19.8 mm (24% length) at the rostral end, 26.2 mm (32% length) at the rear of the tooth row, and 40.4 mm (50% length) at the apex of the coronoid process. Therefore, the dentary is much deeper relative to its length in *Hualianceratops* than in *Yinlong*. A relatively deep dentary is a derived feature that occurs in *Psittacosaurus* [29, 33] and some derived neoceratopsians such as *Protoceratops* [36] and *Leptoceratops* [15] (Fig 8).

The tooth row is medially inset from the lateral surface of the dentary to form a shallow buccal emargination. The emargination is not sharply defined. A row of nine subcircular nutrient foramina is present immediately dorsal to the emargination. The lateral surface is strongly textured, as in *Xuanhuaceratops* [3], the basal neoceratopsian *Archaeoceratops* [5], and pachycephalosaurs [37], but unlike *Yinlong* and *Psittacosaurus* [33] which have smooth, untextured dentaries. In ventral view, the rostroventral ends of each dentary curves medially to meet its partner at the midline forming a spout-shaped symphysis as in all ornithischians [19].

A large subround external mandibular fenestra is present at the junction of the dentary, surangular, and angular. The dentary then projects caudodorsally to form the rostral portion of the low coronoid eminence. The dentary meets the surangular along a straight, caudodorsally oriented suture, and the angular along a similarly oriented but shorter suture. The dentary does not project caudally below the angular. On its medial surface, the dentary has an extensive contact with the splenial (see below), as well as a short contact with the rostralmost part of the prearticular.

#### Surangular

The rostral part of the surangular is preserved with the right mandible (Fig 7). It is thin and slightly bowed laterally, and forms the rostroventral margin of the external mandibular fenestra. The ventral part of the lateral surface is strongly textured like the dentary. This differs from *Yinlong* [1], where the surangular lacks external texturing (Fig 8A and 8C). The dorsal edge of the surangular is expanded mediolaterally to form a bar-like dorsal margin that is oriented nearly horizontally in its preserved rostral portion, and reaches to the highest point of the dentary on the coronoid eminence.

#### Angular

The rostral portion of the right angular is preserved with the right mandible, and forms the rostroventral margin of the external mandibular fenestra. The surface of the angular is strongly textured on both its lateral and ventral surfaces. The ventral portion of the angular expands medially to form a thick and robust ventral mandibular margin. The medial surface of the angular is partially covered by the caudal splenial.

#### Splenial

Both splenials are exposed in medial view, but are missing their caudalmost ends (Fig 7C and 7D). The plate-like splenial is applied to the medial surface of the dentary, surangular, and angular. The tapered rostral end extends nearly to the dentary symphysis. The ventral margin of the splenial extends caudally, paralleling the ventral margin of the dentary. The dorsal margin expands rapidly caudodorsally until it covers nearly the entire medial dentary at its apex near the coronoid process of the dentary (44 mm deep at this point). A large opening on the dorsal margin has broken margins and is likely due to damage. Behind the apex of the splenial, the caudal margin of the splenial plunges sharply caudoventrally to 22 mm deep at the base of the caudal ramus. This margin also forms the rostral and ventral margins of an oval internal mandibular fenestra. Behind the internal mandibular fenestra, the dorsal and ventral margins of the splenial begin to converge as the element tapers caudally and covers the internal surface of the ventral angular.

#### Prearticular

A thin sliver of the rostral prearticular is preserved along the medial surface of the mandible (Fig 7C and 7D). The rostral prearticular is dorsoventrally narrow, and extends up the rear of the splenial, terminating close to its apex, to form the dorsal and caudal margin of the internal mandibular fenestra.

### Dentition

No premaxillary teeth are preserved. The apical half of an unworn tooth crown is exposed in the right maxilla, approximately in the middle of the toothrow (Fig 2C and 2D). The exposed crown is triangular with four large, tabular denticles along the mesial and distal carinae, flanking a similarly sized apical denticle. Denticular ridges are absent; damage to the surface of the tooth precludes determining whether an apical ridge was present.

Several teeth are present in the left dentary, but all are poorly preserved. They are triangular in lingual view, expanding at the base and tapering to their apex. Weak ridges are present on the lingual surface, as in basal ceratopsians *Yinlong* and *Chaoyangsaurus*. No prominent apical ridge is present, in contrast to neoceratopsians (e.g., *Archaeoceratops*) and *Psittacosaurus* [31].

### Postcranial skeleton

The skull material was found associated with a partial skeleton, although most of this material is in very poor condition and little information can be gleaned at present.

Two co-ossified sacral centra are present (Fig 9A and 9B) but more were certainly present; one is nearly complete and the other is fragmentary. The centrum length exceeds its width, which is greater than the height. Fragments of the proximal right tibia and fibula are present, but severely compressed and poorly preserved. The shaft of the left fibula is fractured but otherwise well-preserved. It is slender and straight, robust at the proximal end and narrows distally. The articular ends are not preserved.

Part of the left pes is well preserved in articulation and partially prepared from the block (Fig 9C); it includes the distal ends of metatarsals (MT) I and II, the complete second digit, and parts of the third and fourth digit.

The distal end of MT I is visible in ventral view. Its distal end is slightly curved medially, and the shaft is semicircular in cross section. The ventral surface is flattened to slightly concave. The distal condyles are partially damaged, although the lateral condyle appears more prominent than the medial. The distal end of MT II is more robust than that of MT I. The distal condyles of MT II are dorsoventrally expanded and well separated from one another by a deep ginglymus. A deep tendon insertion pit is centered on the lateral surface of the exposed condyle.

The phalanges of digits II-IV are well preserved. Both phalanges are preserved for digit II. Digit III preserves the distal end of the first phanlanx, and both distal phalanges. Digit IV is represented by the distal end of the proximal phalanx, and the three more distal phalanges. All phalanges are taller than mediolaterally wide, have well-developed ginglymi, and posses deep tendon insertion pits on the lateral surfaces of all phalanges. As in most ornithischians, the phalanges decrease in overall size from digit II through digit IV.

The ungual phalanges of digits II and III are missing their tips, but that of digit IV is complete. All unguals are slightly downcurved, mediolaterally compressed, and have an elongate sulcus on their medial and lateral surface. In all respects they resemble those of *Yinlong*.

### Phylogenetic analysis results

**Fig 10:**
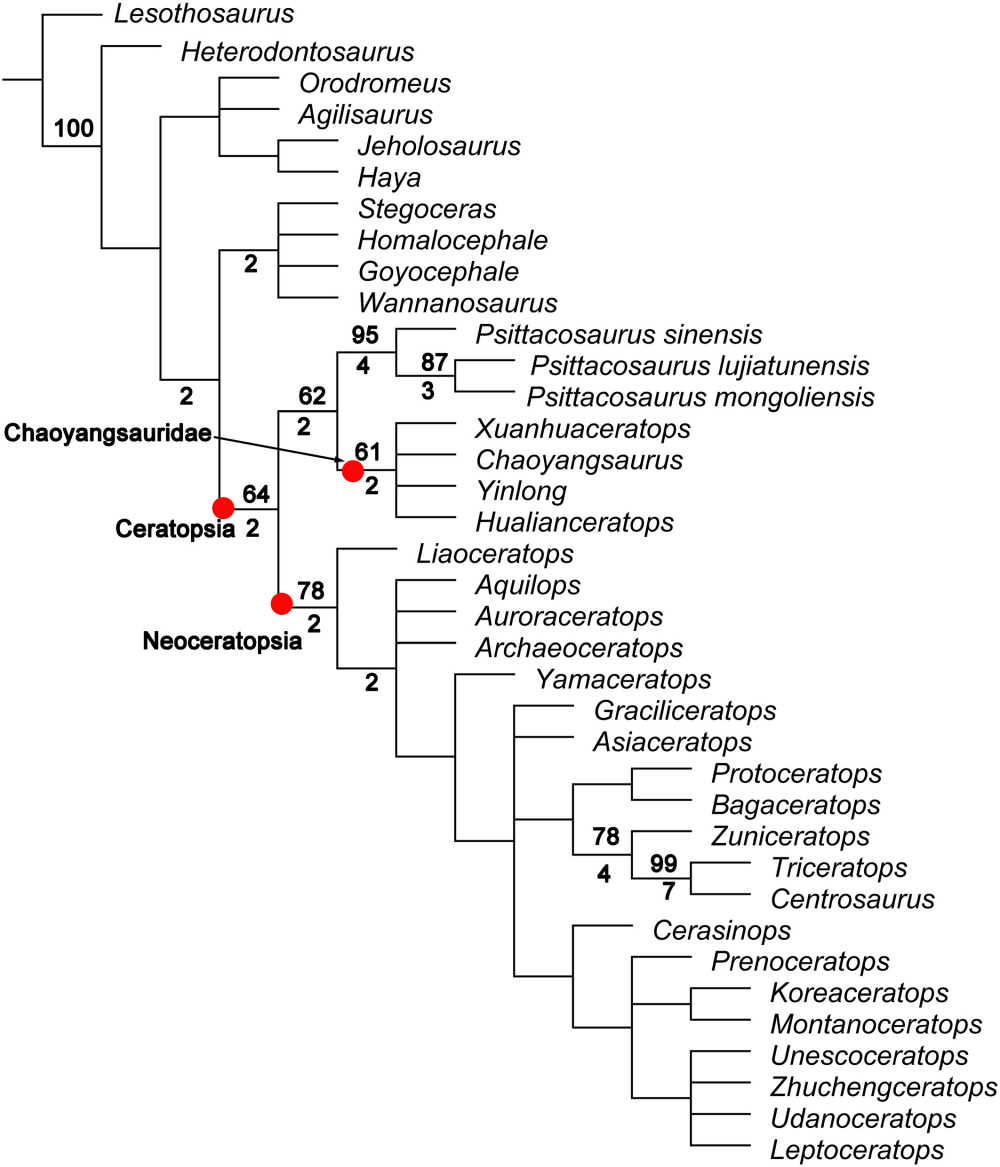
Strict consensus tree of 216 most parsimonious trees produced by analysing a data matrix of 37 taxa and 210 characters. Bremer support (values above 1) is shown below and to the left of nodes, and bootstrap values are labeled to the left and above the nodes (values above 50%).

The analysis recovered 216 most parsimonious trees of 461 steps each (Consistency Index = 0.49; Retention Index = 0.78). The strict consensus tree is shown in Fig 10 along with bootstrap and Bremer support values. This analysis places *Yinlong* and *Hualianceratops* within a monophyletic Chaoyangsauridae, along with *Chaoyangsaurus* and *Xuanhuaceratops*, as suggested by Morschhauser [11] and Han [38] (Fig 10). No unambiguous synapomorphies support this clade, but nine characters (61, 62, 67, 70, 76, 109, 119, 138, 143) under either ACCTRAN or DELTRAN optimize to this node in the strict consensus. Nevertheless, the bootstrap value for this node is 61, while Bremer support is 2, similar to the support for the *Psittacosaurus*-Chaoyangsauridae clade that has six unambiguous synapomorphies. Relationships among the four chaoyangsaurids are unresolved. This is likely due, in part, to the preponderance of missing data and non-overlapping data in *Hualianceratops* (81% missing data), *Xuanhuaceratops* (78%), and *Chaoyangsaurus* (50%). Until more complete materials of these taxa are discovered, their relationships to one another may remain impossible to decipher.

Despite their co-occurrence in the Shishugou Formation and general similarity, *Yinlong* and *Hualianceratops* are supported as sister taxa only in half (108) of the 216 MPTs (maximum parsimony trees). In the MPTs where they are sister taxa, they are united by a single character: a prominent sulcus on the ventral quadratojugal for articulation with the jugal (character 70). A sulcus is present but weakly developed in *Chaoyangsaurus*, and absent in *Xuanhuaceratops*. Additionally, *Yinlong* and *Hualianceratops* are the only ceratopsians known to have a dorsally flat, laterally and caudally expanded squamosal (character 61). However, this character also occurs in pachycephalosaurs and the condition is unknown in either *Chaoyangsaurus* or *Xuanhuaceratops* (though it is absent in *Psittacosaurus*); it may therefore prove to be a synapomorphy of Marginocephalia. Because these characters are ambiguously distributed among the MPTs, neither can support a sister-group relationship between *Yinlong* and *Hualianceratops*, and thus the two taxa cannot be considered congeneric. Other MPTs have *Yinlong* closer than *Hualianceratops* to the other chaoyangsaurids based on character 74; or *Hualianceratops* sister to *Xuanhuaceratops* based on character 120, and *Chaoyangsaurus* sister to these two taxa based on character 144.

**Fig 11:**
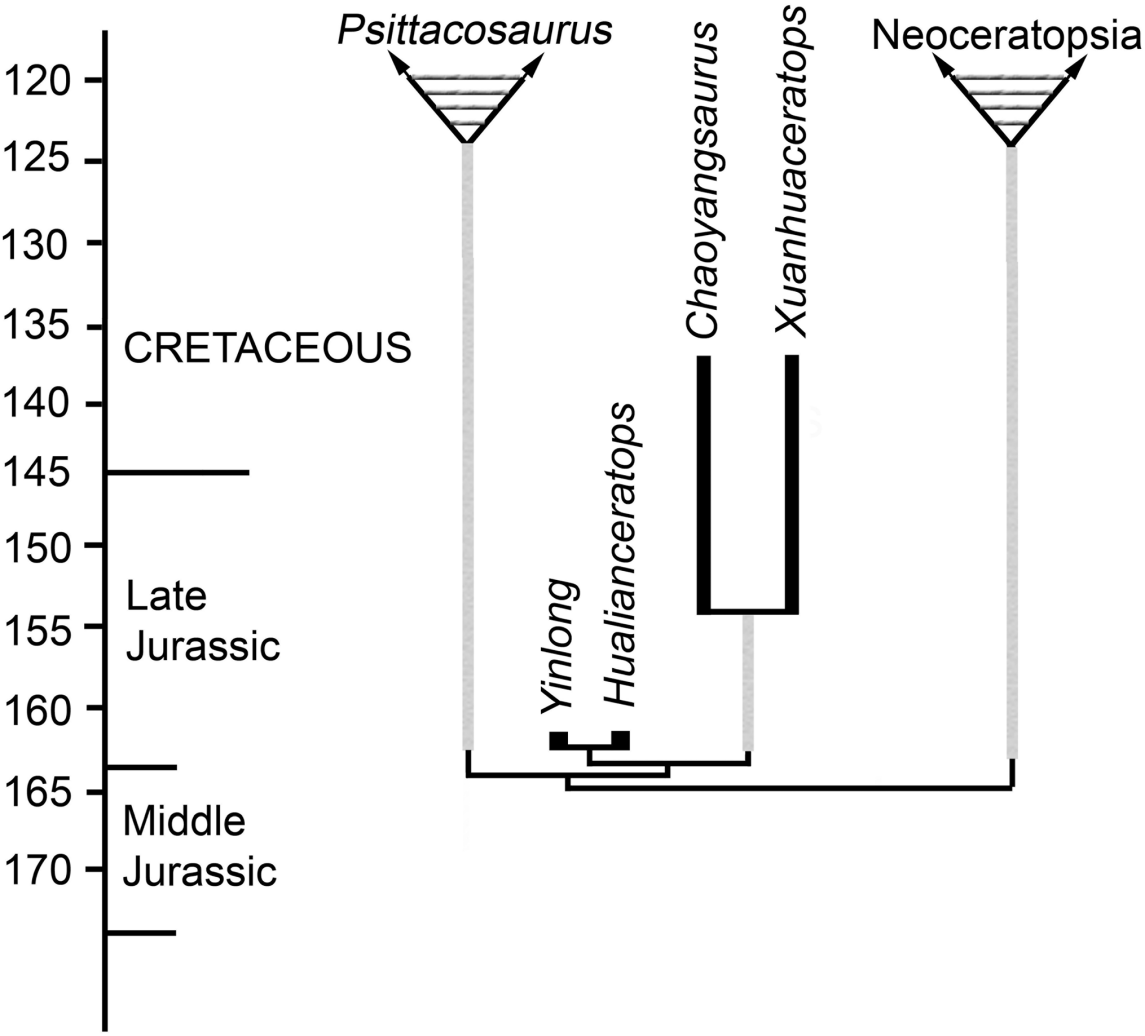
Ghost lineages implied by this phylogenetic analysis. Ghost lineages (gray lines) implied by the stratigraphic distributions of taxa and the most parsimonious trees. Thick black lines indicate single occurrences, their length reflects uncertainty in dating. One of the phylogenies implying the fewest lineages is shown. Geochronology from [41].

The phylogenetic analysis suggests a sister-taxon relationship between Chaoyangsauridae and *Psittacosaurus* that is weakly supported with a bootstrap value of 62 and Bremer support of 2, and in some trees two steps longer *Psittacosaurus* is sister-taxon to Neoceratopsia. Six unambiguous synapomorphies support this clade, including a short preorbital region less than 40% the length of the skull (character 5), the length of rostral to the caudal edge of maxillary less than 55% of the length of the skull (character 7), the infratemporal ramus of the jugal is longer than the infraorbital ramus (character 46), the jugal infratemporal process is strongly arched laterally (character 48), the infratemporal process of the jugal extends caudally along the ventral margin of the quadratojugal (character 49), and the surangular length is more than 50% of the total mandibular length (character 131). Two of these features relate to the relatively short snout, and three concern the morphology of the infratemporal portion of the jugal. The sixth feature, a relatively long surangular, is recognized here for the first time. This relationship suggests an early divergence between *Psittacosaurus* (late Barremian-Albian) [39, 40] and chaoyangsaurids (Late Jurassic) prior to the Late Jurassic, and thus a long ghost lineage (about 38 Ma) for *Psittacosaurus* (Fig 11).

This analysis weakly supports a basal split between a *Psittacosaurus* + Chaoyangsauridae clade and Neoceratopsia, a novel result. This removes the chaoyangsaurids from Neoceratopsia following the definition of the group by Sereno [42]: The most inclusive clade including *Triceratops horridus* but not *Psittacosaurus mongoliensis*. The monophyly of Neoceratopsia in this analysis is supported by a bootstrap value of 78 and Bremer support of 2, and *Psittacosaurus* groups with Neoceratopsia only in trees three or more steps longer than the MPTs. The relationships found in our analysis imply that this basal split in the Ceratopsia occurred prior to the Late Jurassic (Fig 11). Currently, the earliest known neoceratopsian is *Liaoceratops* from the lowermost part of the Early Cretaceous Yixian Formation of China, where it co-occurs with *Psittacosaurus* [4,40]. This phylogeny suggests a long, undiscovered ghost lineage for Neoceratopsia through the Late Jurassic.

## Discussion and Conclusion

*Hualianceratops* (IVPP V18641) represents the second species of basal ceratopsian present in the upper part of the Shishugou Formation at the Wucaiwan locality. Though *Yinlong* possesses a number of autapomorphies, the incompleteness of the *Hualianceratops* material does not allow all of these characters to be evaluated. While two characters have been recognized that are uniquely shared by these taxa (a deep sulcus on the ventral surface of the quadratojugal for articulation with the jugal, and a squamosal with a flat dorsal surface that expands both laterally and caudally), neither unambiguously define a sister-group relationship between these taxa (see above). *Hualianceratops* is distinct from *Yinlong* in possessing the following characters: a prominent dorsal process on the infratemporal ramus of the jugal, a robust quadrate with an expanded rostral margin above the quadratojugal facet, an expansive quadratojugal facet, a deep notch on the ventral jugal wing of the quadrate, a shallow sulcus between the quadrate condyles, and strongly rugose sculpturing on the lateral surface of the dentary. None of these characters occur in individuals of *Yinlong* of any size, suggesting they are not ontogenetically dependent [14].

*Yinlong downsi* shares some derived feature with both *Psittacosaurus* and neoceratopsians [14]. Interestingly, *Hualianceratops* shares more derived characters with *Psittacosaurus* than with basal neoceratopsians. This includes the divergent quadratojugal process and the flattened ventral surface of the jugal, the caudodorsally curved quadrate head, the deep and short dentary. However, the large antorbital fossa, preserved squamosal and sculpture lateral surface of most bones are quite different from that of *Psittacosaurus*. Additionally, the wide jugal-postorbital bar is more like basal neoceratopsians.

The age of the two Shishugou species within the dating error for the beginning of the Oxfordian [41] coupled with the most parsimonious phylogenies imply that at least five lineage of ceratopsians were present at the beginning of the Late Jurassic (Fig 11), including the two Shishugou species. The grouping of *Psittacosaurus* with chaoyangsaurids (Fig 11) implies long ghost lineages for *Psittacosaurus* and Neoceratopsia. By comparison, if there are no morphological constraints on the phylogeny then only two ceratopsian lineages are minimally necessary at the beginning of the Oxfordian, the two Shishugou species. Furthermore, all of the alternative MPTs indicate at least three lineages of chaoyangsaurids were present (assuming the autapomorphies of the two Shishugou taxa debar them from being direct ancestors to any other taxa). Three lineages are implied when the two Shishugou taxa are sister-taxa with a *Chaoyangsaurus-Xuanhuaceratops* clade or when the former are paraphyletic with the latter, but four lineages are implied when *Chaoyangsaurus* and *Xuanhuaceratops* are paraphyletic to a *Yinlong*-*Hualianceratops* clade. The presence of at least five lineages at the beginning of the Late Jurassic contrasts with the previous published analyses indicating only a minimum of two lineages at this time [1, 9], *Yinlong* and all other ceratopsians, and prior to 2006 no ceratopsians were known from the beginning of the Late Jurassic. In any case, this phylogeny implies that ceratopsian phylogenetic diversification was well established by the beginning of the Late Jurassic.

## Supporting Information

S1 File Supplementary figure and character list used in the phylogenetic analysis of Ceratopsia. (DOC)

S2 File Data matrix and character codings used in the phylogenetic analysis of Ceratopsia. (NEX)
