# Correction: A New Taxon of Basal Ceratopsian from China and the Early Evolution of Ceratopsia

**DOI:** 10.1371/journal.pone.0148689

**Published:** 2016-02-01

**Authors:** Fenglu Han, Catherine A. Forster, James M. Clark, Xing Xu

There is an error in the caption title for Fig 6, “The squamosal of *Yinlong downsi* (IVPP V18641).” Please see the complete, correct Fig 6 caption here.

**Fig 6:**
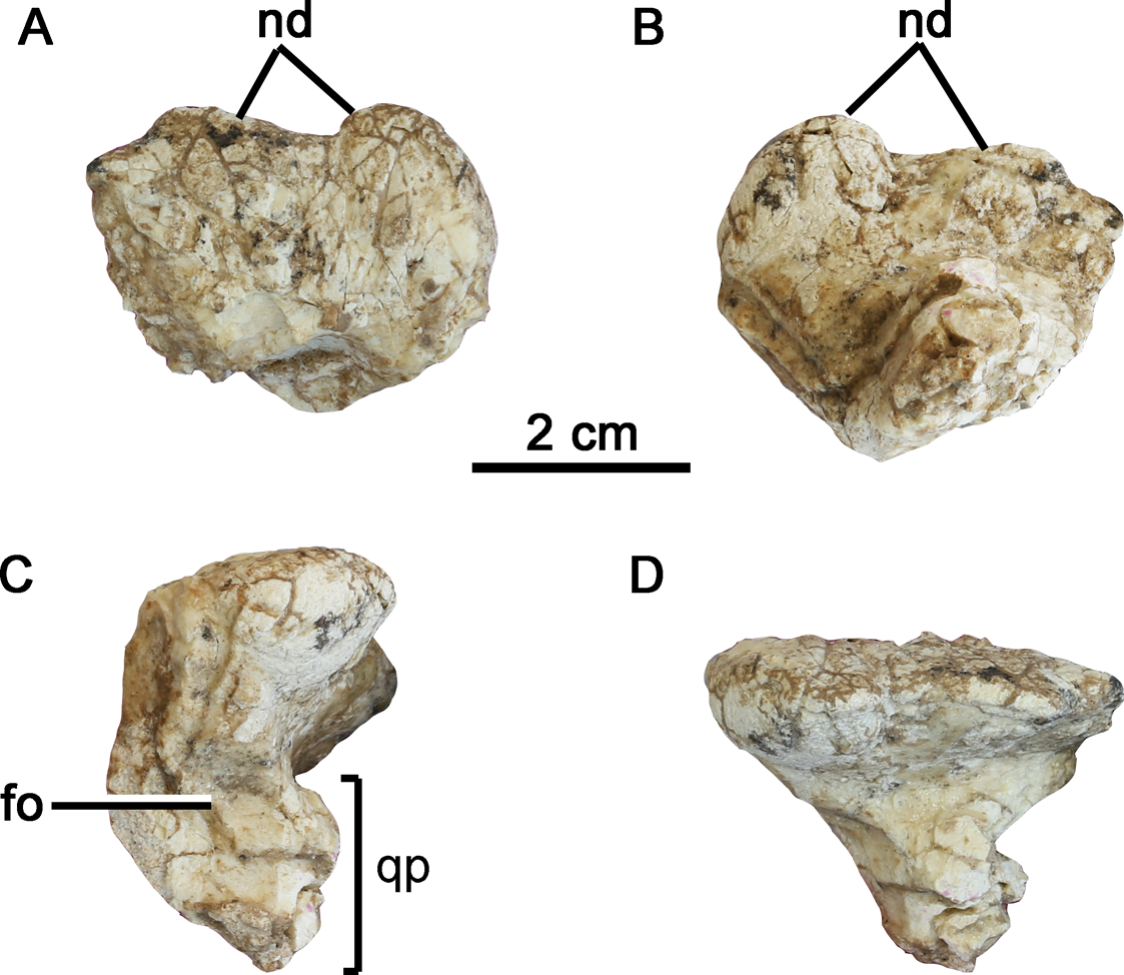
The squamosal of *Hualianceratops wucaiwanensis* (IVPP V18641). (A) dorsal view, (B) ventral view, (C) left lateral view, (D) caudal view. Abbreviations: fo, fossa on the lateral surface; nd, nodes on the caudal margin; qp, stalked quadrate process.
