# Supplementary material for: A New Taxon of Basal Ceratopsian from China and the Early Evolution of Ceratopsia
Source: PLoS One. 2015 Dec 9;10(12):e0143369. doi: 10.1371/journal.pone.0143369 (PMC4674058; doi:10.1371/journal.pone.0143369)
Supplement: S1 File — (DOC) [file pone.0143369.s001.doc]

Supplementary Electronic Material for:

A New Taxon of Basal Ceratopsian from China and the Early Evolution of Ceratopsia

Fenglu Han^1,2*^, Catherine A. Forster^3^ , James M. Clark^3^, Xing Xu^2^

^1^ School of Earth Sciences, China University of Geosciences, Wuhan, China,

^2^ Key Laboratory of Evolutionary Systematics of Vertebrates, Institute of Vertebrate Paleontology and Paleoanthropology, Chinese Academy of Sciences, Beijing, China,

^3^ Department of Biological Sciences, The George Washington University, Washington, District of Columbia, United States of America

* Corresponding author

E-mail: [hfl0501@163.com](mailto:hfl0501@163.com)

This file includes:

1. Supplementary figure
2. Character lists
3. Character changes in basal ceratopsians
4. References
5. **Supplementary figure**


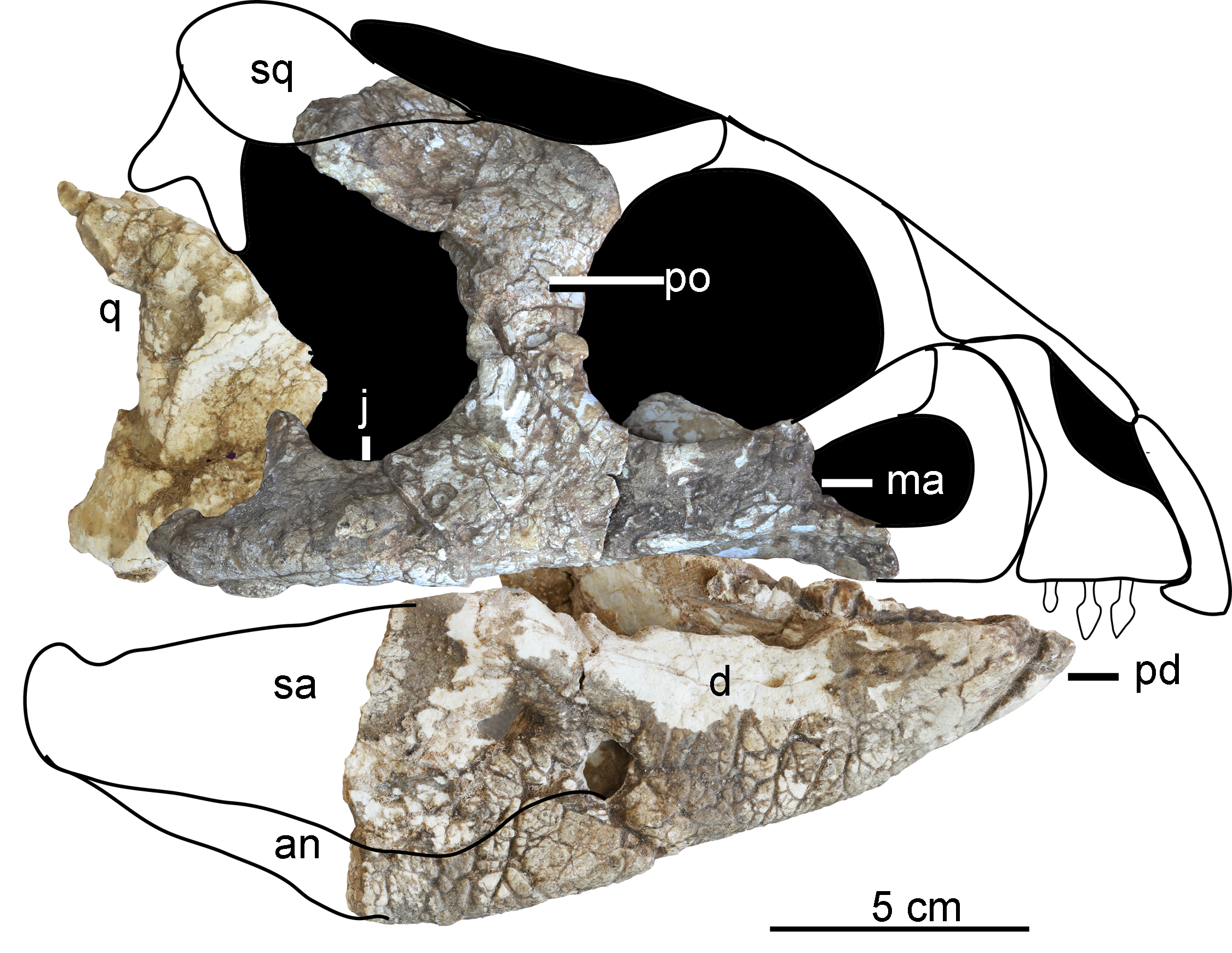


**Figure S1. Reconstructed skull of the holotype specimen of *Hualianceratops wucaiwanensis* (IVPP V18641).** Abbreviations: an, angular; d, dentary; j, jugal; ma, maxilla; pd, predentary; po, postorbital; q, quadrate; sa, surangular; sq, squamosal

**2. Character List**

To assess the systematic position of *Hualianceratops*, a new character list and matrix was compiled and analyzed. Our data matrix was mainly based on those of Ryan et al. [1] and Farke et al. [2], which were in turn modified from the matrices of Makovicky and Norell [3], Makovicky [4], and Xu et al. [5], and Lee et al. [6]. However, these matrices contain few characters germane to the basalmost ceratopsians. To rectify this situation, characters were added from the matrices of Sereno [7, 8], Norman et al. [9], Weishampel et al. [10], Xu et al. [11], and Butler et al. [12]. Multistate characters 19, 20, 70, 98, 128, 146, 171, 174 and 178 were ordered. **The modified characters are marked with “#” and new characters are marked with “*”.**

1. #Skull length (rostral to the caudal end of the quadrate condyle): 15% or less of trunk length (0) or more than 20% of trunk length (1). [Makovicky and Norell, 2006, (1); Butler et al., 2008, (2)]
2. Head shape in dorsal view: ovoid, widest at orbit (0) or triangular, widest across temporal region (1). [Makovicky and Norell, 2006, (2)]
3. #Orbit diameter: more than 25% of skull length (0) or less than 20% of skull length (1). [Makovicky and Norell, 2006, (3)]
4. Supratemporal fenestra length relative to skull length: short, less than 20% skull length (0) or elongated, more than 25% skull length (1). [Xu et al., 2006, (58)]
5. #Length of preorbital region relative to skull length: more than 40% (0) or less than 35% (1) the length of the skull. [Makovicky and Norell, 2006, (4); Butler et al., 2008, (1)]
6. *Skull, rostral margin (rostral margins of the rostral and maxilla): sloped caudodorsally (0) or nearly vertical (1).
7. *Skull length, length from the rostral of the skull to the caudal end of the maxilla relative to the length of skull (rostral to the caudal end of the quadrate condyle): more than 60% (0) or less than 55% (1).
8. #Premaxilla, level of alveolar margin relative to maxillary alveolar margin: below or even with maxilla (0) or raised above maxilla (1). [Makovicky and Norell, 2006, (5)]
9. Rostral bone: absent (0) or present (1). [Makovicky and Norell, 2006, (6)]
10. *Rostral bone, shape of triturating margin: straight (0) or strongly bowed downwards (1).
11. Rostral bone, ventral (buccal) process: absent (0) or present (1). [Makovicky and Norell, 2006, (7)]
12. Rostral bone, sharp keel on midline: absent, convexly rounded (0) or present (1). [Makovicky and Norell, 2006, (8)]
13. Premaxilla, vaulted palate: absent (0) or present (1). [Makovicky and Norell, 2006, (9)]
14. Premaxilla–prefrontal contact: absent (0) or present (1). [Makovicky and Norell, 2006, (11)]
15. #Premaxilla-lacrimal contact: absent (0) or present (1). [Butler et al., 2008, (7)]
16. Premaxilla, convex buccal process rostral to maxillary tooth row formed by premaxilla or premaxilla and maxilla: absent (0) or present (1). [Makovicky and Norell, 2006, (12)]
17. Premaxilla-maxilla, shape of alveolar margin: relatively straight in ventral view, tooth rows converge rostrally (0), sinuous in ventral view, with premaxillary palatal region flaring widely rostral to maxillary tooth row (1). [Makovicky and Norell, 2006, (13)]
18. Nasal, location of rostral end (internarial bar): above (0) or far rostral to the external naris (1). [Makovicky and Norell, 2006, (14)]
19. Nares, position of ventral border of external nares relative to orbit: significantly below (0), at the level (1) or significantly above (2) lower rim of the orbit. [Xu et al., 2002, (100)] ordered
20. Nares, position of ventral border of external nares: significantly below (0), at the level of (1) or significantly above (2) lower rim of the infratemporal fenestra. [Makovicky and Norell, 2006, (16)] ordered
21. Premaxilla, large narial fossa rostroventral to naris: absent (0) or present (1). [Makovicky and Norell, 2006, (17)]
22. Nasal horn: absent (0) or present (1). [Makovicky and Norell, 2006, (18)]
23. Nares, narial fossa, maximum length: shorter than (0) or twice as long as maximum rostrocaudal diameter of the orbit (1). [Sereno, 1999, 3(14)]
24. Nasals, midline depression: absent (0) or present (1). [Makovicky, 2010, (137)]
25. Maxilla, edentulous maxillary rostral margin, length: short, less than 2 tooth widths (0) or long, more than 4 tooth widths (1). [Sereno, 1999, 4(57)]
26. Maxilla-maxilla contact, exposure on palate: absent, covered ventrally by vomers (0) or present (1). [Makovicky and Norell, 2006, (21)]
27. Maxilla, alveolar margin: straight (0) or ventrally convex (1) in lateral view. [Makovicky and Norell, 2006, (22)]
28. *Maxilla, ratio of the width across the rostral end to that across the caudal end of the tooth row: more than 45% (0) or below 40% (1).
29. Antorbital fossa: present (0) or absent (1). [Makovicky and Norell, 2006, (23)]
30. #Antorbital fossa: large, more than 20% the length of the maxilla (0) or small, strongly reduced to less than 10% of the maxilla (1). [Makovicky and Norell, 2006, (23)]
31. Antorbital fossa margins: all sharply defined (0) or rostral margin poorly delineated (1) [Xu et al., 2006, (53)]
32. *Antorbital fossa, shape: rostrocaudal length exceeds dorsoventral depth (0) or rostrocaudal length equal to or shorter than depth (1).
33. *Antorbital fossa, location of ventral margin relative to orbit: the same level (0) or set well below the ventral margin of the orbit (1).
34. Maxilla, accessory fenestra between naris and antorbital fossa: absent (0) or present (1). [Makovicky, 2010, (143)]
35. Maxilla, eminence or tubercle on the rim of the buccal emargination of the maxilla near the junction with the jugal: absent (0) or present (1). [Makovicky and Norell, 2006, (24)]
36. Frontal, surface adjacent to parietal: flat (0), depressed (1) or invaginated by fontanelle (2). [Makovicky and Norell, 2006, (51)]
37. Palpebral attachment: articulates with prefrontal (0) or fused to orbital margin (1). [Makovicky and Norell, 2006, (25)]
38. #Palpebral, length: more than 80% of the width of the orbit (0) or short, less than 50% of the width of the orbit (1). [Weishampel and Heinrich, 1992, (9); Liu, 2004, (39)]
39. Palpebral, shape in dorsal view: rod-shaped (0) or platelike with wide base (1). [Butler et al., 2008, (31)]
40. Jugal–lacrimal contact: short (0) or extensive (1). [Makovicky and Norell, 2006, (26)]
41. Jugal horn on the infratemporal ramus: absent (0) or present (1). [Makovicky and Norell, 2006, (27)]
42. Jugal (or jugal–epijugal) ridge dividing the lateral surface of the jugal into two planes: absent (0) or present (1). [Butler et al., 2008, (38)]
43. Orientation of the jugal horn: laterally directed (0) or ventrally directed (1). [Makovicky and Norell, 2006, (27)]
44. Jugal, relative depths of suborbital and subtemporal rami: sub temporal ramus at least twice the depth of the infraorbital rams and tapers caudoventrally (0) or rami equal in depth or sub temporal rams slightly deeper (1). [Makovicky and Norell, 2006, (28)]
45. Jugal, bifurcated contact with quadratojugal: present (0) or absent (1). [Makovicky, 2010, (136)]
46. *Jugal, relative lengths of the infraorbital and infratemporal rami: infraorbital longer than or equal to infratemporal (0) or infratemporal longer (1).
47. *Jugal, morphology of the ventral margin of the infratemporal process: mediolaterally narrow (0) or ventrally flattened and mediolaterallly expanded (1).
48. *Jugal, infratemporal process: gently bowed laterally (0) or strongly arched laterally (1).
49. *Jugal-quadratojugal contact: the infratemporal process of the jugal contacts only the rostral margin of the quadratojugal (0) or the infratemporal process of the jugal extends caudally along the ventral margin of the quadratojugal, broadly overlaps quadratojugal (1).
50. Jugal-postorbital-angular, sculpted ornamentationon lateral surface: absent (0) or present (1). [Butler et al., 2008, (41)]
51. Jugal–postorbital bar, width broader than infratemporal fenestra: absent, narrow (0) or present (1). [Butler et al., 2008, (42)]
52. Epijugal ossification: absent (0) or present (1). [Makovicky and Norell, 2006, (29)]
53. Epijugal ossification, position: along caudodorsal margin of jugal (epijugal trapezoidal) (0) or capping distal end of jugal (epijugal conical) (1). [Makovicky and Norell, 2006, (30)]
54. Postorbital, horns: absent (0) or present (1). [Makovicky and Norell, 2006, (31)]
55. #Postorbital, shape: with well-defined squamosal and jugal processes (0) or sub-triangular and platelike, lacking well-defined processes (1). [Makovicky and Norell, 2006, (32)]
56. Postorbital, rounded and convex dorsal margin that overhangs the lateral margin of the supratemporal fenestra (0) or with concave dorsal shelf bordering supratemporal fenestra (1). [Makovicky and Norell, 2006, (33)]
57. Postorbital, contribution to infratemporal fenestra: present (0) or absent, excluded by jugal-squamosal contact (1). [Makovicky and Norell, 2006, (34)]
58. Postorbital, if excluded from infratemporal fenestra: narrowly excluded from margin (0), jugal–squamosal contact very wide and postorbital situated far from fenestra (1). [Makovicky and Norell, 2006, (34)]
59. #Infratemporal fenestra, maximum width: more than 20% of skull length (0) or less than 15% of skull length (1). [Makovicky and Norell, 2006, (35)]
60. Squamosal, postquadratic process: absent (0) or present (1). [Makovicky and Norell, 2006, (36)]
61. *Squamosal, dorsal surface flat and expanded laterally and caudally: absent (0) or present (1).
62. *Squamosal, depression on lateral surface continuous with the caudodorsal infratemporal fenestra, insertion point for the M. *adductor mandibulae externus superficialis* muscle: extends caudally behind the quadrate head (0) or restricted to the rostroventral portion of the squamosal, does not extend across the dorsal margin of the quadrate head (1).
63. Squamosal, shape of postorbital process: single ramus (0) or deeply bifurcate (1). [Makovicky and Norell, 2006, (37)]
64. Squamosal, direction of the caudal margin : rostromedially (0) or caudomedially directed, squamosal contributes to lateral portion of frill margin (1). [Makovicky and Norell, 2006, (38)].
65. Squamosal-postorbital bars in dorsal view: parallel (0) or caudally divergent (1). [Makovicky and Norell, 2006, (39)]
66. Squamosal, glenoid set on a distinct, long and robust quadrate process: absent (0) or present (1). [Xu et al., 2006, (100)]
67. Squamosal, enlarged tubercle row on the caudal squamosal: absent (0) or present (1). [Butler et al., 2008, (73)]
68. #Quadratojugal, compression: mediolaterally compressed (0) or transversely expanded and triangular in coronal section (1). [Makovicky and Norell, 2006, (40)]
69. #Quadratojugal, slender rostroventral prong articulating with jugal: absent (0) or present (1). [Makovicky and Norell, 2006, (40)]
70. *Quadratojugal, sulci on the ventral surface for articulation with the jugal: absent (0), present but weak (1) or present but prominent (2). Ordered
71. Quadrate, shape of shaft in lateral view: rostrally convex (0) or straight (1). [Makovicky and Norell, 2006, (41)]
72. *Quadrate, rostral margin above quadrate wing: transversely narrow (0) or transeversely expanded, round and thickened (1).
73. Quadrate, maximum rostrocaudal width relative to height: broad, more than 25% (0) or narrow less than 20% (1). [Sereno, 1999, 4(66); Liu, 2004, (75)]
74. #Quadrate, inclination of articular surface (caudal view) of condyles: horizontal (0) or ventrolaterally inclined (1). [Sereno, 1999, 2(14)]
75. Quadrate (paraquadratic) notch in quadratojugal wing: present (0) or absent (1). [Liu, 2004, (80)]
76. *Quadrate, deep V-shaped sulcus separates the condyles: absent, sulcus is shallow (0) or present (1).
77. Palatine, elongate parasagittal process of the palatine: absent (0) or present (1). [Makovicky and Norell, 2006, (42)]
78. Ectopterygoid, exposure in palatal view: present (0) or absent (1). [Makovicky and Norell, 2006, (43)]
79. Ectopterygoid, contact with jugal: present (0) or absent (1). [Makovicky and Norell, 2006, (44)]
80. Pterygopalatine foramen (modified suborbital fenestra): large, (0) or very small, greatly reduced (1). [Makovicky and Norell, 2006, (45)]
81. Pterygoid, distinct ridge on caudoventral margin of mandibular process: absent (0) or present (1). [Makovicky and Norell, 2006, (46)]
82. Pterygoid–maxilla contact at caudal end of tooth row: absent (0) or present (1). [Makovicky and Norell, 2006, (47)]
83. Pterygoid, prominent caudal midline process covering or partially covering body of basisphenoid and basioccipital: absent (0) or present (1). [Makovicky and Norell, 2006, (48)]
84. Pterygoid mandibular process: long, extending well below maxillary tooth row (0) or short, terminates at or above maxillary tooth row (1). [Makovicky and Norell, 2006, (49)]
85. Pterygoid, mandibular process composition: jointly by pterygoid and ectopterygoid (0) or formed only by pterygoid (1). [Makovicky and Norell, 2006, (50)]
86. Pterygoid, vomeral process: rostrally oriented (0) or dorsally oriented (1). [Xu et al., 2006, (62) ]
87. Parietal-squamosal, extended caudally as distinct frill/shelf that overhangs the occipital condyle: absent (0) or present (1). [Makovicky and Norell, 2006, (52)]
88. Parieto-squamosal frill relative to basal skull length: less than 50% (0) or more than 70% (1). [Makovicky and Norell, 2006, (52)]
89. Parieto-squamosal frill fenestration: absent (0) or present (1). [Makovicky and Norell, 2006, (54)]
90. Parietals, distinctive indentation on midline of the caudal parietals: present (0) or absent (1). [Makovicky and Norell, 2006, (55)].
91. Parietal, sharp sagittal crest: absent, flat or gently convex (0) or present, distinct, narrow and tall (1). [Makovicky, 2010, (138)]
92. Parietal, width: subequal to (0) or much wider than (1) the frontal skull roof. [Sereno, 2000:53]
93. Episquamosal and epiparietal ossifications: absent (0) or present (1). [Makovicky and Norell, 2006, (56)]
94. Basioccipital, participation in foramen magnum: present (0) or absent (1). [Makovicky and Norell, 2006, (57)]
95. Exoccipital, participation in occipital condyle: forms less than one-third of occipital condyle (0) or forms at least half of occipital condyle (1). [Makovicky and Norell, 2006, (57)]
96. #Basioccipital, longitudinal keel below condyle: absent (0) or present (1). [Xu et al., 2006, (95)]
97. #Basioccipital, extent of contribution to basal tubera: basioccipital contribution restricted, does not extend ventrally, basisphenoid contribution to tubera can be seen in caudal view (0) or basioccipital expanded ventrally to form part of basal tubera, basisphenoid contribution cannot be seen in caudal view (1). [Xu et al., 2006, (99)]
98. Basipterygoid process, orientation: rostrolateral (0), ventral (1) or caudoventral (2) when braincase is oriented with dorsal margin of occipital condyle horizontal. [Makovicky and Norell, 2006, (59)] ordered
99. Basioccipital, tubera shape: rostrocaudally compressed (0) or thickened and everted ventrolaterally (1). [Makovicky and Norell, 2006, (60)]
100. Exoccipital, exits for cranial nerves X–XII: three (0) or two (1). [Makovicky and Norell, 2006, (62)]
101. Exoccipital–quadrate contact: absent, separated by squamosal (0) or present (1). [Makovicky and Norell, 2006, (63)]
102. Paraoccipital process, minimum depth relative to total length: depth 50% or more of the length (0) or 40% or less of the length (1). [Makovicky and Norell, 2006, (64)]
103. Supraoccipital participatation in dorsal margin of foramen magnum: present (0) or absent, excluded by exoccipitals (1). [Makovicky and Norell, 2006, (65)]
104. Supraoccipital, orientation of caudal face: rostrodorsally inclined (0) or vertical (1). [Makovicky and Norell, 2006, (66)]
105. #Supraoccipital, shape: taller than wide, triangular (0) or wider than tall, trapezoidal (1) [Makovicky and Norell, 2006, (67)]
106. #Predentary, distinct dorsally curved tip: absent, dorsal margin is straight (0) or present (1). [Makovicky and Norell, 2006, (68); Butler et al., 2008, (94)]
107. Predentary, rostral end (in dorsal view): rounded (0) or pointed (1). [Makovicky and Norell, 2006, (69); Butler et al., 2008, (92)]
108. Predentary length relative to dentary toothrow length: less than or equal to half of dentary toothrow (0) or two-thirds or more of dentary toothrow (1). [Makovicky and Norell, 2006, (70)]
109. Predentary, opposition to premaxilla: predentary short, caudal premaxillary teeth oppose rostral dentary teeth and predentary (0) or long, roughly equal in length to the premaxilla, premaxillary teeth only oppose predentary (1). [Butler et al., 2008, (91) ]
110. Predentary buccal margin, shape: narrow and sharp (0), a rounded, beveled edge (1) or a broad, grooved, triturating edge (2). [Makovicky and Norell, 2006, (71)]
111. Predentary, contact on dentary: no distinct facets for the reception of the predentary on dentary (0) or dentary grooved dorsally for reception of the lateral process of the predentary (1) or bears large pit for reception of the lateral process of the predentary (2). [Makovicky and Norell, 2006, (72)]
112. Predentary, bifurcation of ventral process: absent (0) or present (1). [Butler et al., 2008, (95)]
113. Dentary, length of symphysis measured parallel to long axis of dentary: short, 25% or less (0) or long, 30% or more (1) dentary length. [Makovicky and Norell, 2006, (73)]
114. #Dentary, diastema of at least one tooth width between predentary and first dentary tooth: absent (0) or present (1). [Makovicky and Norell, 2006, (74)].
115. #Dentary, length of diastema: short, equal to one or two alveoli (0) or long, equal to four or more alveoli (1). [Makovicky and Norell, 2006, (74)].
116. #Ventral margin of dentary, shape in lateral view: straight to slightly bowed/sinuous (0) or strongly bowed ventrally (1). [Makovicky and Norell, 2006, (75)]
117. Dentary, ventral flange: absent (0) or present (1). [Makovicky and Norell, 2006, (76)]
118. #Dentary, shallowest height relative to the whole rostrocaudal length in lateral: less than 25% (0) or more than 50% (1). [modified from Xu et al., 2006, (64)]
119. #Dentary, dorsal and ventral margin orientations: subparallel (0) or converge rostrally more than 20% of depth (1). [Butler et al., 2008, (99)]
120. Dentary, development of distinct rostroventral chin behind symphysis: absent (0) or present (1). [Ryan et al., 2012, (150)]
121. Dentary, prominent medial expansion at the center of the tooth row formed by wide Meckelian groove separating tooth-bearing part of the jaw from external surface: absent (0) or present (1). [Makovicky and Norell, 2006, (77)]
122. Dentary, sculptured lateral surface: absent (0) or present (1). [Makovicky and Norell, 2006, (78)]
123. Dentary-prearticular contact on medial surface of mandible: absent (0) or present (1). [Makovicky and Norell, 2006, (79)]
124. Splenial, shape of caudoventral end: tapered (0) or bifurcated (1). [Makovicky and Norell, 2006, (80)]
125. Dentary, coronoid process, notch along caudal margin for insertion of the surangular: absent (0) or present (1). [Makovicky, 2010, (145)]
126. Dentary, coronoid process, size of notch: wide (0) or constricted, deep and narrow (1). [Makovicky, 2010, (146)]
127. Coronoid process, apical rostral expansion: absent (0) or present (1). [Makovicky and Norell, 2006, (81)]
128. Coronoid process position, relative to caudal end of toothrow: arises from main axis of dentary caudal to tooth row (0), set lateral to tooth row, end of tooth row medial to rostral margin of coronoid process (1) or set lateral to tooth row, end of tooth row level with caudal edge of coronoid process (2). [Makovicky and Norell, 2006, (82)] ordered
129. Coronoid bone shape: narrow, subequal depth throughout (0) or with lobate, highly expanded dorsal end that is much deeper than ventral end that slots between splenial and dentary (1). [Makovicky and Norell, 2006, (83)]
130. #Dentary, coronoid process, inclination: steeply inclined caudodorsally (>40 degrees) (0) or vertical (1) [Ryan et al., 2012, (151)].
131. #Surangular, length: less than 40% (0) or more than 50% (1) of the total mandible length. [Xu et al., 2006, (65)]
132. Surangular, prominent process rostroventral to glenoid: absent (0) or present (1) [Makovicky, 2010, (139)]
133. Surangular, distinct lateral ridge on surangular that overhangs the angular: absent (0) or present (1). [Makovicky and Norell, 2006, (84)]
134. Surangular, shape of lateral surface: flat or only slightly convex (0) or with pronounced laterally convex curvature (1). [Makovicky and Norell, 2006, (85)]
135. Surangular, process forming lateral wall to glenoid cotyle: absent (0) or present (1). [Makovicky and Norell, 2006, (86)]
136. Angular, small lateral tubercles along ventral rim below glenoid : absent (0) or present (1) [Makovicky and Norell, 2006, (87)]
137. * Surangular, dorsal margin of concavity on angular, if present: concavity grades smoothly into surangular surface (0), or distinct dorsal margin of concavity formed by ridge on surangular (1).
138. Angular, shape of lateral surface: flat or slightly convex (0) or raised ridge along caudoventral margin with lateral surface above ridge distinctly concave (1). [Makovicky and Norell, 2006, (88)]
139. *Angular, orientation of ventral margin: horizontal (0) or extends caudodorsally to more closely approach the glenoid (1).
140. Surangular, long rostroventral process intervening between rostrodorsal angular and caudal dentary: absent (0) or present (1). [Makovicky and Norell, 2006, (89)]
141. External mandibular fenestra on dentary-surangular-angular boundary: present (0) or absent (1). [Butler et al., 2008, (104) ]
142. Mandibular glenoid: narrow and flush with medial margin of surangular (0) or distinctly medially expanded relative to medial margin of surangular (1). [Makovicky and Norell, 2006, (90)]
143. Articular, medial surface expanded below glenoid into a semicircular process: absent (0) or present (1). [Makovicky and Norell, 2006, (91)]
144. Retroarticular process: present (0) or absent (1). [Makovicky and Norell, 2006, (92); Butler et al., 2008 (107)]
145. Premaxilla, teeth: present (0) or absent (1). [Butler et al., 2008, (111)]
146. #Premaxilla, tooth number: three or more teeth (0), two (1) or one (2) [Makovicky and Norell, 2006, (93)] ordered
147. Premaxillary tooth carinae: present, denticles may be present on some or all teeth (0) or absent (1). [Makovicky and Norell, 2006, (94)]
148. Premaxillary teeth, crown shape: recurved (0) or straight (1). [Sereno, 2000, (Ceratopsian 7)]
149. Premaxillary teeth, size of largest teeth: equivalent to (0) or much larger than (1) maxillary teeth. [(Xu et al., 2006, (73)]
150. Cheek teeth, number of roots: one (0) or two (1). [Makovicky and Norell, 2006, (95)]
151. Cheek teeth, spacing: present, between bases of crowns (0) or absent, teeth closely appressed, spaces between crowns eliminated (1). [Makovicky and Norell, 2006, (96)]
152. #Cheek teeth, occlusion: at an oblique angle (0) or at a vertical angle (1). [Makovicky and Norell, 2006, (97)]
153. Dentary teeth, horizontal shelf on the lateral surface: absent (0) or present (1). [Makovicky and Norell, 2006, (97)]
154. #Cheek teeth, median primary ridge size: low and wide (0) or prominent, narrow and distinct (1). [Makovicky and Norell, 2006, (98)]
155. Maxillary/dentary crown, height relative to width: subequal to (0) or 1.5 times (1) the maximum crown width. [Sereno 1999, 4(69)]
156. Maxillary/dentary primary ridge, position: near center of the crown surface, giving the crown a relatively symmetrical shape in lingual/labial view (0) or distinctly offset, giving the crown an asymmetrical appearance (1). [Butler et al., 2008, (122)]
157. Maxillary teeth, base of primary ridge relative to base of crown/cingulum: confluent with (0) or inset from crown base/cingulum (1). [Makovicky and Norell, 2006, (99)]
158. Cheek teeth, number of replacement teeth: one (0) two or more (1). [Makovicky and Norell, 2006, (101)]
159. Tooth enamel on both lingual and buccal sides of teeth: present (0) or absent, enamel restricted to lateral side of maxillary and medial side of dentary teeth (1). [Makovicky and Norell, 2006, (102)]
160. Dentary, number of alveoli: less than 20 (0) or more than 25 (1). [Makovicky and Norell, 2006, (104)]
161. Cheek teeth, shape of roots: cylindrical (0) or with mesial and distal faces flattened and sometimes slightly grooved for reception of crowns of replacement teeth (1). [Makovicky and Norell, 2006, (105)]
162. Cheek teeth, shape of tooth crown apex: pointed/triangular (0) or rounded (1). [Makovicky and Norell, 2006, (106)]
163. #Cervical vertebrae, number: nine or fewer (0) or ten or more (1). [Butler et al., 2008, (135)]
164. Atlas intercentrum shape: semicircular (0) or circular (1). [Makovicky and Norell, 2006, (107)]
165. Atlas intercentrum, fusion to odontoid: absent (0) or present (1). [Makovicky and Norell, 2006, (108)]
166. Atlas neurapophyses, fusion to intercentrum/odontoid: absent (0) or present (1). [Makovicky and Norell, 2006, (109)]
167. Axis, neural spine shape: low spine-like process (0) or tall and hatchet- shaped process (1) [Makovicky and Norell, 2006, (110)]
168. Axis, neural spine caudal extent: short, extends caudally no more than half way across the succeeding cervical centrum (0) or long, extending caudally to the caudal end of the succeeding cervical centrum (1). [Makovicky and Norell, 2006, (111)]
169. #Cervical vertebrae 1-3, fusion of centra: absent (0) or present (1). [Sereno, 2000, (42); Makovicky and Norell, 2006, (112)]
170. Cervical vertebrae, ventral keels on centra: present (0) or absent (1). [Makovicky, 2010, (147)]
171. Dorsal vertebrae, number: 14 or fewer (0), 15 (1) or 16 or more (2). [Butler et al., 2008, (137)] ordered
172. . #Dorsal vertebrae, mid-dorsal neural spines: short and rectangular, height and craniocaudal length subequal (0) or height more than twice craniocaudal length (1). [Norman, 2002, (41); Liu, 2004, (192)]
173. Dorsal vertebrae, zygapophysesal articular surfaces: flat (0) or with tongue and grooves articulations (1). [Makovicky and Norell, 2006, (113)]
174. Sacral vertebrae, number (including sacrodorsal vertebrae): five (0) or six (1), seven (2) or eight or more (3). [Makovicky and Norell, 2006, (114)] ordered
175. Sacrum and sacral ribs, shape in dorsal view: parallel to slightly concave sides (0) or convex sides (1). [Makovicky and Norell, 2006, (115)]
176. Sacral ribs, caudal sacral ribs are considerably longer than rostral sacral ribs: absent (0) or present (1). [Butler et al., 2008, (140)]
177. #Caudal vertebrae, neural spines on proximal caudals: caudodorsally inclined (0) or vertical (1). [Makovicky and Norell, 2006, (116)]
178. Caudal vertebrae, height ratio of neural spine on mid-caudals to associated centrum: less than or equal to 2 (0), 2.1-3 (1), 3.1-4 (2), more than 4.1 (3). (Lee et al., 2011, 134) ordered
179. Caudal vertebrae, height of the neural spine is greater than the length of the chevron for any given caudal vertebra: absent, chevrons are longer (0) or present (1). [Lee et al., 2011, (136)]
180. Caudal vertebrae, distalmost caudals: cylindrical centra lacking neural spines and chevrons (0) or neural spines and chevrons persist to the end of tail (1). [Makovicky and Norell, 2006, (117)]
181. Chevrons, lobate distal expansions: present (0) or absent (1). [Makovicky and Norell, 2006, (118)]
182. Clavicles, ossified: absent (0) or present (1). [Makovicky and Norell, 2006, (119)]
183. Scapula, shape in sagittal view: bowed laterally (0) or relatively straight (1). [Makovicky and Norell, 2006, (120)]
184. *Scapula, blade orientation relative to long axis of glenoid: blade at an angle to glenoid (0) or almost perpendicular to glenoid (1). [Makovicky and Norell, 2006, (121)]
185. Coracoid, large lateral process (coracoid tubercle) for origin of deltoid musculature near confluence of rostral and ventral margins: absent (0) or present (1). [Makovicky and Norell, 2006, (122)]
186. Ulna, size of olecranon process: relatively small (0) or enlarged to one-third or more of ulnar length (1). [Makovicky and Norell, 2006, (123)]
187. Radius, lateral and medial tuberosities along distal half of shaft: absent (0) or present (1). [Makovicky, 2010, (144)]
188. Carpals, number of distal carpals: three or more (0) or two or fewer (1). [Makovicky and Norell, 2006, (124)]
189. Manus-pes relative lengths: manus much smaller than pes (0) or manus and pes subequal in size (1). [Makovicky and Norell, 2006, (125)]
190. Ilium, dorsal margin of preacetabular process and body above acetabulum: narrow, not transversely expanded (0) or transversely expanded to form a narrow but distinct shelf (1). [Butler et al., 2008, (168)]
191. Ilium, preacetabular process, length: longer than (0) or shorter than the postacetabular process (1). [Liu, 2004, (264)]
192. Ilium, preacetabular process expands mediolaterally towards its distal end: absent (0) or present (1). [Butler et al., 2008, (169)]
193. Ilium, eversion of dorsal margin of postacetabular process: absent or weak (0) or prominent (1). [Xu et al., 2006, (92)]
194. *Ilium, depth of blade above acetabulum: dorsoventrally deep, depth more than 60% the width between rostral margin of the pubic peduncle and the caudal margin of ischial peduncle at the base (0) or shallow, depth less than 50% the peduncle-peduncle length (1).
195. Pubis, postpubis length: long, approximately equal in length to the ischium (0) or very short or absent (1). [Butler et al., 2008, (188)]
196. Pubis, shaft of postpubis in cross section: subrounded (0) or mediolaterally flattened and bladelike (1). [Makovicky and Norell, 2006, (126)]
197. Pubis, postpubis orientation: caudoventrally oriented (0) or caudally directed (1). [Makovicky and Norell, 2006, (127)]
198. Pubis, prepubic process, distal expansion: absent, subequal in depth or tapered along length (0) or present, constricted proximal portion followed by a distal expansion (1) [Norman, 2002, (58); Liu, 2004, (282)]
199. Pubis, prepubic process width: Compressed mediolaterally, dorsoventral height exceeds mediolateral width (0) or mediolateral width equal to or exceeds dorsoventral height (1) [Butler et al., 2008, (193)]
200. Pubis prepubic process, length: short, does not reach to the distal end of the preacetabular process of ilium (0) or long, extends to or beyond distal end of preacetabular process of ilium (1). [Butler et al., 2008, (194)]
201. Ischium, tab-shaped obturator process: present (0) or absent (1). [Butler et al., 2008, (184)]
202. Ischium, shaft shape: straight (0) or with dorsally convex curvature (1). [Makovicky and Norell, 2006, (129)]
203. Femur, shape in medial/lateral view: bowed anteriorly along length (0) or straight (1). [Butler et al., 2008, (197)]
204. Femur, fourth trochanter shape: large and pendant (0) or reduced to low eminence (1). [Makovicky and Norell, 2006, (130)]
205. Femur length versus tibia length in adults: shorter (0) or equal or longer (1). [Makovicky and Norell, 2006, (131)]
206. Femur, anterior (extensor) intercondylar groove on distal end of femur: absent (0) or present (1). [Butler et al., 2008, (203)]
207. Pedal phalanges shape: gracile with elongate phalanges (0) or short, all phalanges wider than long (1). [Makovicky and Norell, 2006, (132)]
208. Metatarsals, arrangement: compact, closely appressed to one another along 50–70% of their length, spread distally (0) or contact each other only at proximal ends, spread strongly outwards distally (1). [Butler et al., 2008, (210)]
209. Pedal unguals, shape: pointed (0) or broad and distally rounded (1). [Makovicky and Norell, 2006, (133)]
210. Pedal unguals, width of proximal end relative to width of distal end of the preceding phalanx: equal (0) or wider (1). [Lee et al., 2011, (135)]

**3 character changes in basal ceratopsians**

Original and revised cranial character scores for basal ceratopsians based on our first hand observation. Changes are shown in bold typeface. ? = unknown; “-” inapplicable.

*Yinlong downsi*

|  | 10 | 20 | 30 | 40 | 50 | 60 |
| --- | --- | --- | --- | --- | --- | --- |
| Previous | 11010??11? | 00???0?011 | 00010?0?00 | 0??0000111 | 01-0?????1 | 00-0100-00 |
| This paper | 1101**1010**1**0** | **1**0**1**?**0**0**1**011 | 00010**1**0**0**00 | 0**01**00**1**011**0** | 0**0**-**101011**1 | 00-0**0**00-0**1** |
|  | 70 | 80 | 90 | 100 | 110 | 120 |
| Previous | ??000110-? | 0?100?000? | 00?0?000-1 | 111001120? | ?00?101010 | 100000011 |
| This paper | **10**00**1**110-**2** | 0**0**100**1**000? | 00**1**0**0**000-1 | 11**0**001120? | ?**1**0**0**1010**0**0 | 1**?1**0000**0**1 |
|  | 130 | 140 | 150 | 160 | 170 | 180 |
| Previous | 00?00?-000 | 010000?1?? | 01?1010110 | 00000?0000 | ?0?00??10? | 11??010??? |
| This paper | 00**0**00**0**-000 | 010000**0**1**10** | 01**1**1010110 | 00000-0000 | **0**0**0**00**00**10? | 11**00**010**000** |
|  | 190 | 200 | 210 |  |  |  |
| Previous | 0????????0 | 110?1??011 | 1?00?0?000 |  |  |  |
| This paper | 0?**01**?**0000**0 | 110**1**1**00**011 | 1**0**00**0**0**0**000 |  |  |  |

*Chaoyangsaurus*

|  | 10 | 20 | 30 | 40 | 50 | 60 |
| --- | --- | --- | --- | --- | --- | --- |
| Previous | 110?1??11? | 001??00?12 | 0???0????? | ????1????? | 00-1?????1 | ?0??????0? |
| This paper | 110?1**110**1**0** | **?**01??0**1**?1**?** | 0???0?**00**?? | ????1**1**???? | 00-**?**?**1111**1 | ?0??????0? |
|  | 70 | 80 | 90 | 100 | 110 | 120 |
| Previous | ???????0-? | ???0?????0 | 0000?0???? | ???00?1?0? | ?????11010 | ?110010010 |
| This paper | ???????0-**1** | ???0?**1**???0 | 00**1**0?0???? | ???00?1?0? | ?????**00**0**0**0 | ?**?**100**0**0010 |
|  | 130 | 140 | 150 | 160 | 170 | 180 |
| Previous | 01??0-?000 | 100000?0?? | 0111020100 | 0000010000 | 0000000000 | ?????????? |
| This paper | 0**0**??0-**0**000 | 100000**011**? | **?**111020100 | 00000**-**0000 | 00**?**0000000 | ?????????? |
|  | 190 | 200 | 210 |  |  |  |
| Previous | ????0????? | ?????????? | ?????????? |  |  |  |
| This paper | ????**?**????? | ?????????? | ?????????? |  |  |  |

*Xuanhuaceratops*

|  | 10 | 20 | 30 | 40 | 50 | 60 |
| --- | --- | --- | --- | --- | --- | --- |
| Previous | ?????????? | ??????0??? | ?????????? | ?????????? | ?????????1 | 0????????? |
| This paper | ?????????? | ??????**?**??? | ?????????? | ?????????? | ????????**1**1 | 0????????? |
|  | 70 | 80 | 90 | 100 | 110 | 120 |
| Previous | ?????????? | ???1?????? | ?????????? | ?????????? | ?????????? | 1?100100?0 |
|  | ?????????**0** | ???1?**1**???? | ?????????? | ?????????? | ?????????? | 1?100**0**00?0 |
|  | 130 | 140 | 150 | 160 | 170 | 180 |
| Previous | ?1???-???? | ??0000?0?? | ?11102?1?0 | 00?0??0000 | 00?00???**0**0 | ??0??????? |
| This paper | ?1???-???? | ??0**?**00**011**? | ?11102?1?0 | 00?**?**??0000 | 00?00????0 | ??0??????? |
|  | 190 | 200 | 210 |  |  |  |
| Previous | ??110????? | ?????????? | ???0??0??? |  |  |  |
| This paper | ??**0**10????? | ?????????? | ???0??0??? |  |  |  |

*Liaoceratops*

|  | 10 | 20 | 30 | 40 | 50 | 60 |
| --- | --- | --- | --- | --- | --- | --- |
| Previous | ?1010??11? | 1111101011 | 0001010?11 | 1??0100??0 | 11001????0 | 10-0101000 |
| This paper | ?1010**000**1**0** | 111110101**0** | 0001010**100** | 1**11**0**01**0??0 | 1**0**0**1**1**00001** | 10-0101000 |
|  | 70 | 80 | 90 | 100 | 110 | 120 |
| Previous | ??1111000? | 1?100??000 | 0010101011 | 1001011100 | ?100111010 | 1110001010 |
|  | **01**1111000**0** | 1**0**100**0**?000 | 0010**0**01011 | 100**0**011100 | ?10011**?**010 | 1**0**1000**0**0**0**0 |
|  | 130 | 140 | 150 | 160 | 170 | 180 |
| Previous | 10000?01?0 | 000111?0?0 | 1101010?10 | 0001110?10 | 01???0???? | ?????????? |
| This paper | 10000**-**01?0 | 0001**0**1**0**0**1**0 | **0**101010110 | 000**0**110?10 | 01???**?**???? | ?????????? |
|  | 190 | 200 | 210 |  |  |  |
| Previous | ????0????0 | ?????????? | 1??????0?? |  |  |  |
| This paper | ????0????**?** | ???**1**?????? | 1??????0?? |  |  |  |

*Archaeoceratops*

|  | 10 | 20 | 30 | 40 | 50 | 60 |
| --- | --- | --- | --- | --- | --- | --- |
| Previous | 11010??111 | 1110011011 | 00001?0?10 | 1??0000110 | 11011????1 | 1??01?1010 |
| This paper | 11010**00**111 | 111001101**0** | 00001?0**100** | 1**11**00**1**0110 | 11011**0000**1 | 1??01?**?0**1**?** |
|  | 70 | 80 | 90 | 100 | 110 | 120 |
| Previous | ????110100 | 1?11???0?0 | 0?10?010?? | 11?100110? | 0100111011 | ?110110010 |
|  | **0**???110100 | 1**0**11?**0**?**?**?**?** | **?**?10?010?? | 11?100110? | 0100111011 | ?1101**0**0010 |
|  | 130 | 140 | 150 | 160 | 170 | 180 |
| Previous | 10??0?01?0 | 001110?01? | 1101010100 | 000**1**1?0?00 | ?1?000???0 | 001001?000 |
| This paper | 1**1**??0**-**01?0 | 001110**0**01? | **?**10101**1**100 | 00001**1**0?**?**0 | ?1?**???**??**0**0 | 001**1**01?000 |
|  | 190 | 200 | 210 |  |  |  |
| Previous | ?????????0 | 100?1??011 | 10????0000 |  |  |  |
| This paper | ?????????0 | 1**1**0**1**1??011 | 10????0000 |  |  |  |

**4. References**

1. Ryan MJ, Evans DC, Currie PJ, Brown CM, Brinkman D. New leptoceratopsids from the Upper Cretaceous of Alberta, Canada. Cretaceous Res. 2012;35:69-80.

2. Farke AA, Maxwell WD, Cifelli RL, Wedel MJ. A ceratopsian dinosaur from the Lower Cretaceous of Western North America, and the biogeography of Neoceratopsia. PLoS One. 2014;9(12):e112055.

3. Makovicky PJ, Norell MA. *Yamaceratops dorngobiensis*, a new primitive ceratopsian (Dinosauria: Ornithischia) from the Cretaceous of Mongolia. Am Mus Novit. 2006:1-42.

4. Makovicky P. A redescription of the *Montanoceratops cerorhynchus* holotype, with a review of referred material. In: Ryan MJ, Chinnery-Allgeier BJ, Eberth DA, Ralrick PE, editors. New perspectives on horned dinosaurs: The Royal Tyrrell Museum Ceratopsian Symposium: Indiana University Press, Bloomington; 2010. p. 68-82.

5. Xu X, Wang K-B, Zhao X-J, Sullivan C, Chen S-Q. A new leptoceratopsid (Ornithischia: Ceratopsia) from the Upper Cretaceous of Shandong, China and its implications for neoceratopsian evolution. PLoS One. 2010;5(11):e13835.

6. Lee Y-N, Ryan MJ, Kobayashi Y. The first ceratopsian dinosaur from South Korea. Naturwiss. 2011;98(1):39-49.

7. Sereno PC. The evolution of dinosaurs. Science. 1999;284:2137-47.

8. Sereno PC. The fossil record, systematics and evolution of pachycephalosaurs and ceratopsians from Asia. In: Benton M, Shishkin M, Unwin D, Kurochkin E, editors. The age of dinosaurs in Russia and Mongolia. Cambridge: Cambridge University Press; 2000. p. 480-516.

9. Norman DB. On Asian ornithopods (Dinosauria: Ornithischia). 4. *Probactrosaurus* Rozhdestvensky, 1966. Zool J Linn Soc. 2002;136(1):113-44.

10. Weishampel DB, Jianu CM, Csiki Z, Norman DB. Osteology and phylogeny of Zalmoxes (ng), an unusual euornithopod dinosaur from the latest Cretaceous of Romania. J Sys Palaeonotol. 2003;1(2):65-123.

11. Xu X, Forster CA, Clark JM, Mo J-Y. A basal ceratopsian with transitional features from the Late Jurassic of northwestern China. Proc R Soc B. 2006:2135-40.

12. Butler RJ, Upchurch P, Norman DB. The phylogeny of the ornithischian dinosaurs. J Sys Palaeonotol. 2008;6(1):1-40.
